# Post-guidance signaling by extracellular matrix-associated Slit/Slit-N maintains fasciculation and position of axon tracts in the nerve cord

**DOI:** 10.1371/journal.pgen.1007094

**Published:** 2017-11-20

**Authors:** Krishna Moorthi Bhat

**Affiliations:** Department of Neuroscience and Cell Biology, University of Texas Medical Branch School of Medicine, Galveston, Texas, United States of America; ICM, FRANCE

## Abstract

Axon-guidance by Slit-Roundabout (Robo) signaling at the midline initially guides growth cones to synaptic targets and positions longitudinal axon tracts in discrete bundles on either side of the midline. Following the formation of commissural tracts, Slit is found also in tracts of the commissures and longitudinal connectives, the purpose of which is not clear. The Slit protein is processed into a larger N-terminal peptide and a smaller C-terminal peptide. Here, I show that Slit and Slit-N in tracts interact with Robo to maintain the fasciculation, the inter-tract spacing between tracts and their position relative to the midline. Thus, in the absence of Slit in post-guidance tracts, tracts de-fasciculate, merge with one another and shift their position towards the midline. The Slit protein is proposed to function as a gradient. However, I show that Slit and Slit-N are not freely present in the extracellular milieu but associated with the extracellular matrix (ECM) and both interact with Robo1. Slit-C is tightly associated with the ECM requiring collagenase treatment to release it, and it does not interact with Robo1. These results define a role for Slit and Slit-N in tracts for the maintenance and fasciculation of tracts, thus the maintenance of the hardwiring of the CNS.

## Author summary

Early during embryogenesis, the Slit ligand is present only at the midline of the nerve cord. It binds its receptor Robo on growth cones of axons and guides axons such that they form a series of axon tracts called longitudinal tracts on either side of the midline. It has been proposed that Slit regulates axon guidance through a Slit gradient emanating from the midline. Mid-way through embryogenesis, a distinct set of axons begins to cross the midline, forming commissures. Slit from the midline travels along commissural tracts to the longitudinal connectives. The function of Slit in tracts is not known. The Slit protein is processed into a larger N-terminal peptide and a smaller C-terminal peptide. Here, I show that Slit/Slit-N interact with Robo in tracts to maintain the fasciculation and position of axon tracts following axon guidance. In the absence of Slit in mature tracts after their guidance, the tracts de-fasciculate and merge with one another and shift their position towards the midline. This work also shows that Slit and Slit-N are not freely present in the extracellular milieu but associated with the extracellular matrix and both interact with Robo1. Slit-C is bound tightly in the ECM and does not interact with Robo1. These results define a role for Slit in tracts for axon maintenance and fasciculation, thus the hardwiring of the CNS itself.

## Introduction

In the Drosophila embryonic ventral nerve cord, about 20 longitudinal axon tracts traverse up and down the nerve cord to connect all hemisegments on either side of the midline. These tracts are inter-connected across the midline by the commissural tracts, which cross the midline only once and never re-cross. The longitudinal tracts, together with those commissural tracts that cross the midline, form the longitudinal connectives on either side of the midline. Pathfinding of longitudinal and commissural tracts have been studied in detail [1–11]. It is well documented that signaling pathways such as Slit-Robo [1–7] or Netrin-Frazzled [8–11], guide growth cones of these tracts to their synaptic targets. In the absence of these signaling cues, growth cones follow aberrant routes from the very beginning of their journey.

Slit-Robo signaling is the main system that mediates pathfinding of growth cones for the longitudinal tracts [3–7]. In Drosophila, the *slit* gene is transcribed in the midline glia, and the protein is present in the midline glia [1–3], whereas its receptors, Robo1, Robo2, and Robo3 are present in a combinatorial manner in axon growth cones [4–6]. The interaction between Slit and Robo mediates proper projection of growth cones on either side the midline parallel to each other. A loss of function for *slit* early during neurogenesis, for instance, causes the projection of pCC, a pioneering growth cone for the medial longitudinal tract, to head tangentially towards the midline [3, 7]. In older *slit* mutant embryos, all longitudinal tracts are collapsed at the midline. Loss of function for *robo* genes also causes collapsing of tracts at the midline or on to each other [4–7].

While the role of guidance molecules in axon pathfinding has been well-explored, it is less so whether these molecules are also required for maintaining the position of axon tracts. A previous study had explored the role of Slit on the fasciculation of tracts and their spacing in the mouse diaphragm [12], but not much is known if the position of tracts and their fasciculation are maintained within the CNS. By examining a mutation in the enzyme involved in the glycosylation of Slit, we have recently shown that Slit-Robo signaling contributes to the maintenance of axon tracts [13]. This issue of maintenance of tracts has major implications since it is essential for maintaining the integrity of the hardwiring of the nervous system, thus, neural function, or dysfunction later in development or in life.

While Slit is expressed in the midline glia [1–3], Slit is also found in tracts [7, 13, see also 1–3, 14]. We ruled out Slit in tracts as cross-reactivity to the antibody or a background staining and showed that tracts-Slit is transported from the midline to the longitudinal connectives along the commissural tracts [7, 13]. Since both Slit and Robo are present in tracts of the longitudinal connectives, it is most likely that they would interact with each other in tracts to mediate certain function.

The Slit protein is processed into a larger N-terminal peptide and a smaller C-terminal peptide. It has been suggested that the C-terminal peptide interacts with PlexinA1 to mediate commissural axon guidance, and the N-terminal peptide interacts with Robo to mediate guidance of longitudinal tracts [15, 16]. Because of a serendipitous discovery that the expression pattern of Slit is affected in late stage *patched* (*ptc*) mutant embryos, I sought to explore the post-guidance function of Slit in the Drosophila ventral nerve cord using mutations in *ptc*. Ptc was originally identified as a major segmentation gene, but it also regulates neurogenesis both in flies and vertebrates [17–21]. In this study, I report a novel effect of loss of function for *ptc* on Slit in the CNS of older stage embryos. This effect was accompanied by a novel nerve cord defect, which correlated with the changes in the expression of Slit and its localization in tracts. Ptc, together with mutations in another axon guidance molecule, Commissureless (Comm), which is involved in the down-regulation of Robo1 in commissural tracts at the midline [22–24], allowed me to examine the role of Slit in the maintenance of position and fasciculation of tracts. I also found that Robo1 binds to both full-length Slit and Slit-N in tracts but not to Slit-C. Slit and Slit-N are not freely found in the extracellular milieu, but are associated with the ECM. Compared to Slit or Slit-N, Slit-C appears to be more tightly associated with the ECM of axon tracts requiring collagenase treatment to release it. These results reveal novel insights into this important signaling system in the CNS.

## Results

### Loss of function for *ptc* causes a progressive anterior-posterior funneling of axon tracts of the mature embryonic nerve cord

**Fig 1:**
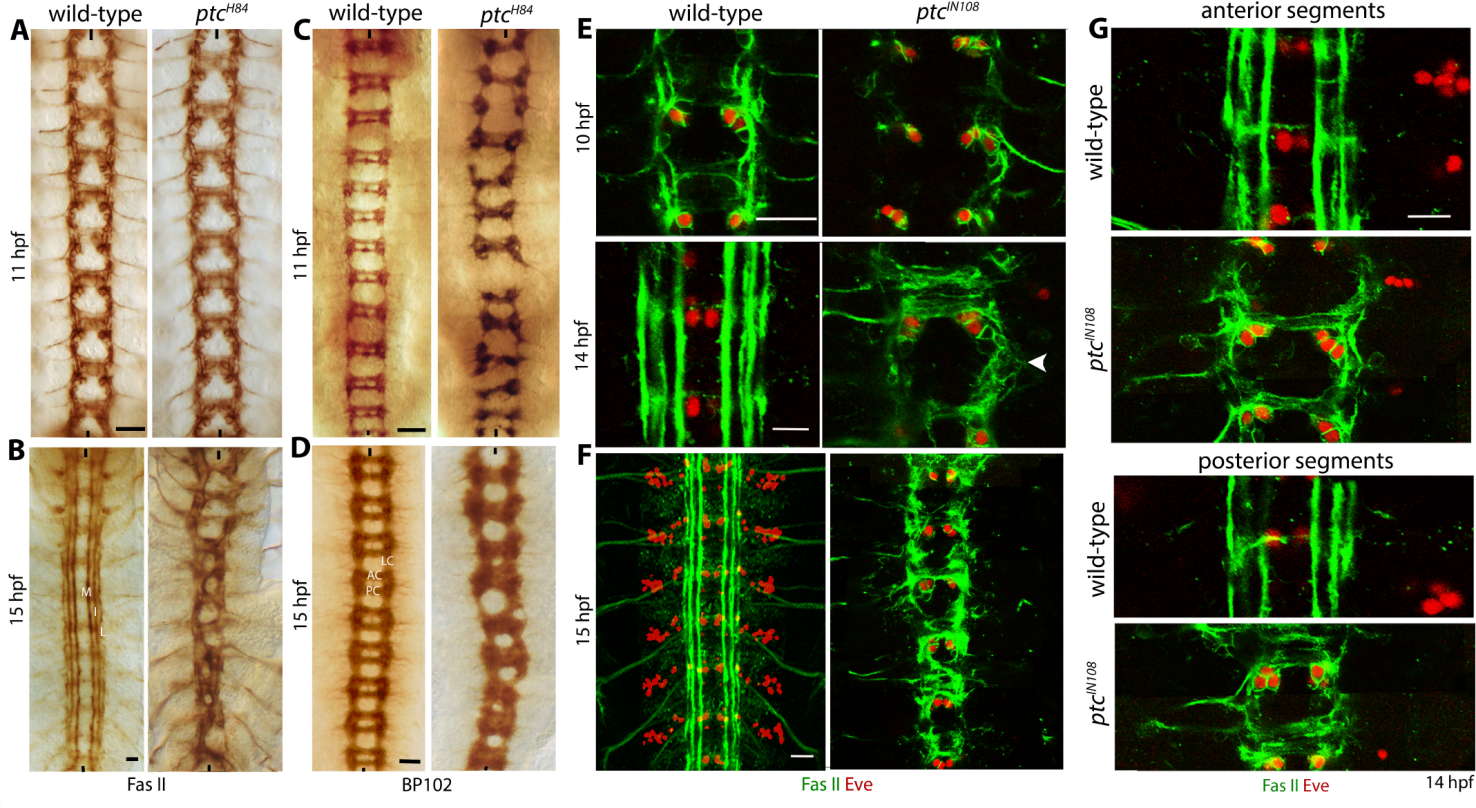
Axon tracts of the ventral nerve cord in *ptc* mutant embryos progressively narrow with age in an anterior-posterior direction. (A-D): Wild-type and *ptc* mutant embryos aged 10 and 15 hours of development were stained with Fas II (A, B) and BP102 (C, D) and analyzed with diamino benzidine (DAB) staining. Fas II stains medial (M), Intermediate (I) and Lateral (L) tracts, which become clearly defined by 15 hours of development. BP102 stains anterior commissure (AC), posterior commissure (PC) and longitudinal connective (LC). LC contains both the longitudinal axons and the portion of commissural axons that crosses the midline. Note the well-defined position of tracts along the nerve cord, parallel to the midline and to each other in wild-type. In older mutant embryos, the tracts progressively shift towards the midline. Scale bar: Panels A and C: 22 μm, panels B and D: 8 μm. (E-F): Wild-type and *ptc* mutant embryos were double stained with Fas II and Even-skipped (Eve) antibodies and analyzed with confocal microscopy. Eve was used as a position-marker. In panel E, 10 hpf and 14 hpf wild-type and *ptc* embryos are shown. While the newly formed individual tracts are well-defined in 10 hpf embryos, the tracts are defasciculated in 14 hpf *ptc* mutant embryos (arrow head). In pane F, 15 hpf embryos are shown. By 15 hours of development, the tracts in *ptc* show the funneling phenotype. In *ptc* mutant embryos axons inappropriately cross the midline, but this defect is related to the misspecification of neuronal identity [20]. Scale bar: Panel E: 20 μm (upper images) and 8 μm (lower images), and Panel F: 8 μm. (G): Defasciculation in the anterior segments is less severe compared to the posterior segments in *ptc* embryos. Wild-type and *ptc* mutant embryos were double-stained for Fas II and Eve. In the anterior segments in *ptc* mutant, the tracts are much better defined compared to the posterior segments. Scale bar: 8 μm. All the images shown are representative from at least N = 3 independent observations unless otherwise stated.

Ptc is involved in multiple developmental and disease processes [17–21]. We have previously reported that misspecification of the identity of neurons that send pioneering axons is responsible for some of the axon guidance defects in *ptc* mutant embryos [20]. A closer examination of the ventral nerve cord in *ptc* loss of function embryos showed a novel nerve cord defect in embryos that were older than 13 hours post fertilization (hpf; at 22 ^0^C). As shown in Fig 1, Fasciclin II (Fas II) and BP102 staining of mutant embryos showed that axon tracts progressively moved towards the midline in the anterior-posterior direction resulting in a funneling phenotype (panels B, D, F, see also Table 1). This funneling defect was seen in 82+/-3.6% (n = 30 embryos, N = 3 independent experiments). The difference in the distance between L-L tracts in *ptc*, but not in wild-type embryos, was statistically significant between the anterior and the posterior regions (P<0.001; S1 Data). This defect was not seen in embryos that were younger than 12–13 hpf (Fig 1A, 1C and 1E; Table 1), but was seen in embryos older than 13 hpf (Fig 1B, 1D and 1F; Table 1). While the fasciculation of longitudinal tracts was more or less normal in younger stage *ptc* mutant embryos (Fig 1E, upper images), in older embryos these tracts were frayed and de-fasciculated with no observable discreet bundles (Fig 1E, lower image, arrowhead). The defasciculation defects were much more pronounced in the posterior segments compared to the anterior segments (Fig 1G). These results suggest that in older *ptc* mutant embryos, tracts not only move towards the midline in the posterior region but also they de-fasciculate.

**Table 1:** Distance between longitudinal tracts and connectives in the anterior and posterior ends in wild-type, *ptc*, and *comm* mutant embryos.

Age	Genotype	Distance Between Tracts			
		Fas II		BP102	
		L—L		C—C	
		Anterior	Posterior	Anterior	Posterior
10–11 hr	wild-type (μm)	28.2 ± 0.7	27.8 ± 0.5	27.8 ± 0.5	26.2 ± 0.8
	*ptc* (μm)	27.8 ± 0.5	28.0 ± 0.9	27.6 ± 0.6	26.2 ± 0.7
15 hr	wild-type (μm)	30.0 ± 0.5	28.3 ± 1.2	30.5 ± 0.7	28.5 ± 0.4
	*ptc* (μm)	29.3 ± 0.4	17.8 ± 0.8	30.1 ± 0.8	17.4 ± 0.6
	*comm* (μm)	58.8 ± 2.9	37.0 ± 2.6	61.8 ± 1.3	43.3 ± 1.4

The distance between tracts across the midline (in μm with standard deviation). The values given are as a mean with Standard deviation compiled from 6 different embryos. M-M is the distance between medial tracts across the midline, L-L, the distance between the two lateral tracts, C-C is the distance between the outer edges of the connectives across the midline. The difference in the distance between tracts in wild-type and *ptc* mutant embryos was statistically significant only in the posterior region of the nerve cord in 15 hpf embryos (P<0.001, two-tailed P-value using the student *t*-test). The distance between I tracts across the midline was not calculated because of the severe tracts defects. The measurements given here for *comm* embryos are for those that show the funneling phenotype (see text). About 10% of *comm* embryos have severe midline defects with holes and breaks, and we have not used such embryos in our analysis.

**Fig 2:**
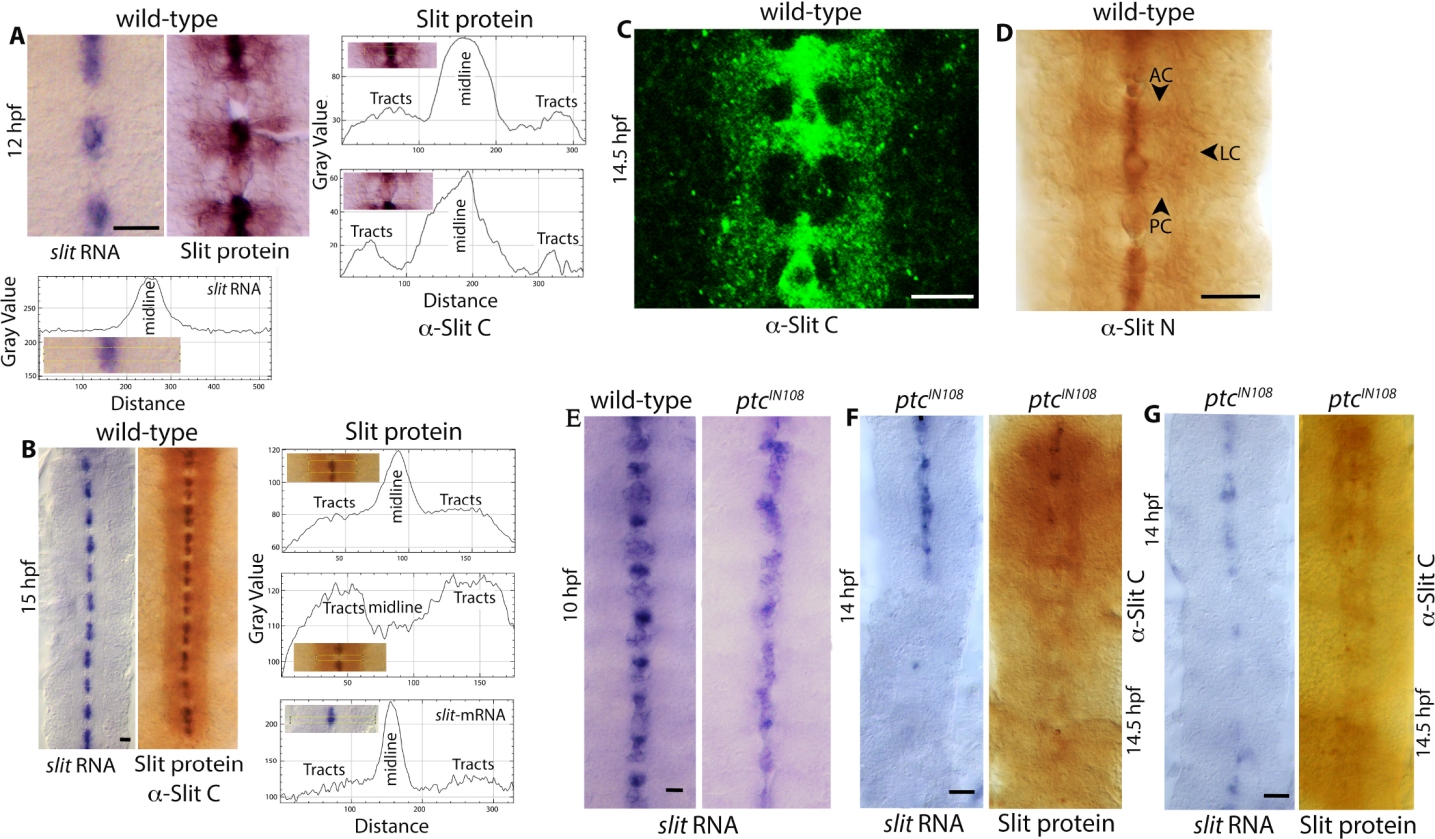
The expression of Slit is progressively lost from the midline and tracts in an anterior-posterior direction in *ptc* mutant embryos. (A, B):Wild-type embryos were examined for *slit* transcription by RNA whole mount in situ (alkaline phosphatase, AP) and Slit protein (anti-Slit-C) by DAB-histochemistry. The image analysis was done using the ImageJ software and shown as expression profile plot. The yellow-rectangle in the inset photomicrograph marks the area of ImageJ analysis. The *slit* transcription is restricted to the midline glia, and the Slit protein is present in midline glia as well as in axon tracts of commissures and longitudinal connectives. Scale bar: Panel A, 10 μm; panel B, 8 μm. (C, D): Wild-type embryos were stained with anti-Slit-C and the signals were detected using a fluorescently labeled secondary antibody and confocal microscopy (C, single section-plane), or stained with anti-Slit-N and detected with DAB-histochemistry (D). Note the presence of Slit in commissures and connectives. AC, anterior commissure; PC, posterior commissure; LC, longitudinal connectives. Scale bar: 8 μm. (E): The expression of *slit* RNA in 10 hpf wild-type and *ptc* mutant embryos. No loss of *slit* expression is detected at this age. Scale bar: 8 μm. (F): The expression of *slit* mRNA and protein in 14 hpf wild-type and 14.5 hpf old *ptc* mutant embryos. The expression (both the mRNA and the protein) is progressively lost from the midline as well as from the tracts in an anterior-posterior direction (compare panel F to panel B). Scale bar: 8 μm. (G): In a minority of *ptc* embryos, loss of Slit expression was less organized, although the posterior region was more affected. In such embryos as well, the narrowing of tracts phenotype correlated with the loss of abundance of Slit in tracts. Scale bar: 8 μm.

The *slit* gene is transcribed in the glial cells at the midline [1, 2; see Fig 2A, 2B and 2E). However, the protein is present in the midline glia as well as in axon tracts of the commissures and connectives (Fig 2A and 2B)[7, 13, see also ref. 1–3, 14]. The Slit in tracts appears to be transported along the commissural tracts, with the protein profile extending from the midline source cells to the longitudinal connectives along the newly forming commissural tracts (Fig 2A). This can be clearly seen with the ImageJ analysis of the staining. ImageJ analysis across an area that has the Slit-positive midline cells and the commissural tracts crossing these midline cells showed a midline peak that continues with a tiny dip before a smaller peak representing Slit in longitudinal tracts on either side of the midline (Fig 2A and 2B). A similar ImageJ analysis across the midline but between the anterior commissure (AC) and the posterior commissure (PC)(between neuromeres) also showed a midline peak and two smaller peaks corresponding to the Slit in longitudinal tracts, but the dip in between the midline and the longitudinal tracts peaks reached the baseline (Fig 2A and 2B). This dip was due to the absence of axon tracts and consequently the absence of any Slit in that region. ImageJ analysis of the *slit-*mRNA staining showed a single peak at the midline (Fig 2A).

In older ~15 hpf embryos, while the mRNA expression was restricted to the midline cells, a clear ladder-like Slit protein in tracts was seen (Fig 2B). The continuous domain of Slit along the midline now was resolved into two distinct domains, one located at the AC and the other at the PC in each neuromere/segment (Fig 2B). Consistent with Slit being spread to the longitudinal tracts along the AC and PC tracts, Slit was seen in AC and PC but not in between (Fig 2B). ImageJ analysis across the midline and at AC or PC showed a midline Slit-peak, and two peaks on either side of the midline corresponding to Slit in longitudinal tracts (Fig 2B). Whereas analysis across the midline but between neuromeres/segments, showed a dip at the midline because of lack of midline Slit, but only two Slit peaks in longitudinal tracts (Fig 2B). The spreading of Slit from the midline to the tracts along the commissural tracts can be seen with immunofluorescent labeling of Slit in ~14.5 hpf old embryos (Fig 2C). The Slit protein was seen strictly along the tracts but not in the areas between AC, PC, and LC, where no tracts are present. A gradient of Slit extending from the midline source to the periphery across the nerve cord was not observed. Staining embryos with an antibody against Slit-N also showed the presence of Slit in tracts and did not reveal any Slit-gradient (Fig 2D). These results show that outside of the midline, Slit is found only in tracts. Consistent with these findings, in embryos mutant for *comm*, where commissures are mostly absent or greatly reduced [23, 24, 7], Slit was present in the midline but mostly absent or greatly reduced in longitudinal connectives [7]. Thus, commissural tracts appear to play the conduit role in moving Slit from the midline to longitudinal connectives.

The expression of Slit in *ptc* mutant embryos was not affected during the early stages of axon guidance [20, see Fig 2E). However, in older *ptc* mutant embryos the expression decayed in a highly specific manner (Fig 2F). The mutant embryos that were 14 hpf or older had *slit* mRNA present in the anterior midline region of the nerve cord, but not in the posterior region (Fig 2F; 83%+/-11% of the embryos, n = 30 embryos/experiment, N = 3 separate experiments). Consistent with this pattern of *slit* RNA expression, in about the same percentage of embryos, the protein at the midline was restricted to the anterior region in these older *ptc* mutant embryos (Fig 2F). Moreover, Slit in tracts also followed the midline pattern with the protein present at higher levels in the anterior region and progressively decreased towards the posterior region (Fig 2F). In the remaining ~17% +/-11% of the embryos, a few *slit-*expressing midline cells could be found in the posterior region or interspersed along the midline (Fig 2G), and about the same percentage of embryos also had Slit protein distributed in a similar fashion in the midline as well as in tracts (Fig 2G). These results indicate that midline is the source of Slit in tracts and the presence of Slit in tracts mirrors Slit expression in the midline.

### The funneling of tracts in late stage *ptc* mutant embryos is due to a corresponding progressive loss of Slit expression

**Fig 3:**
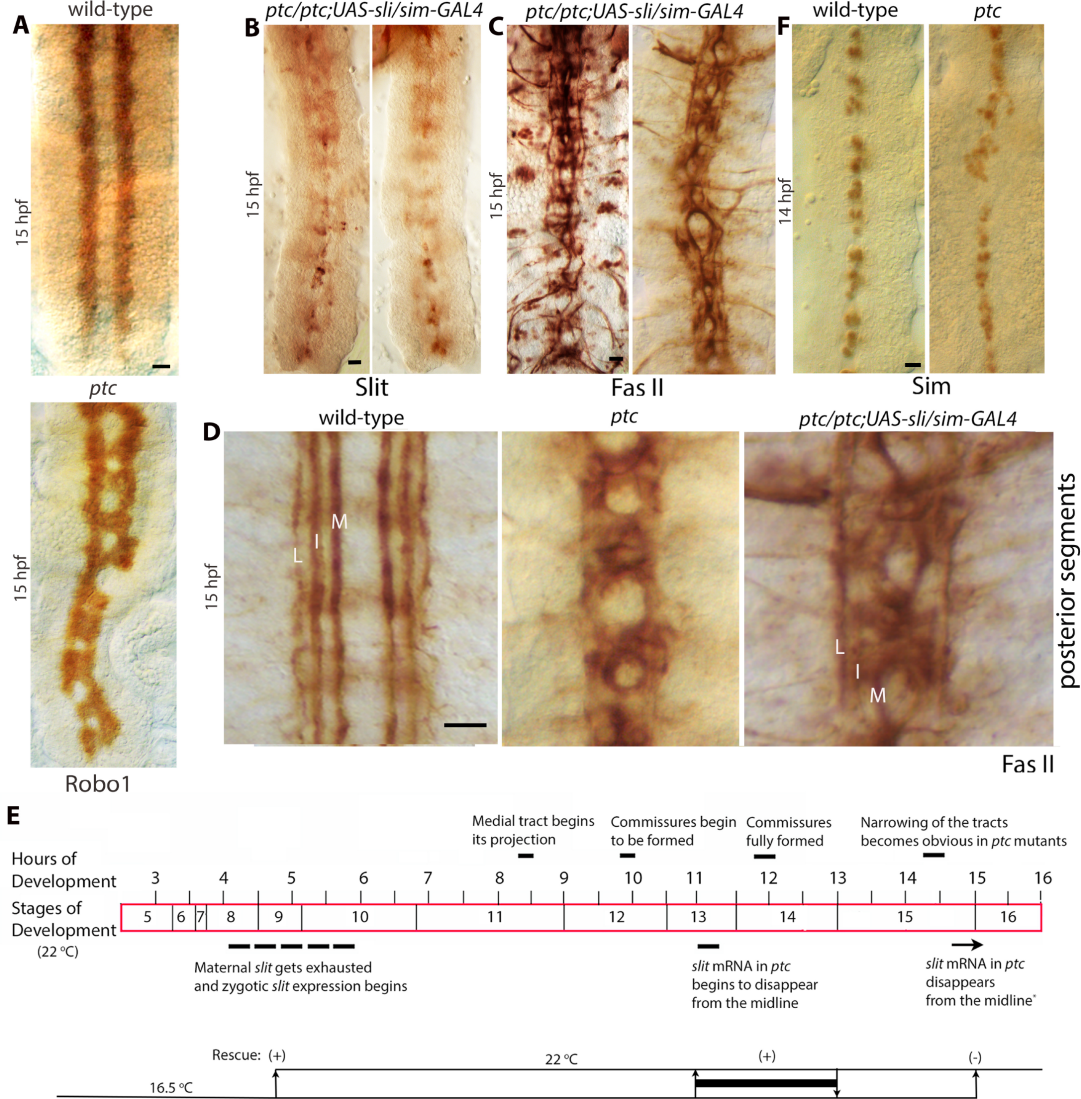
Expression of the *slit* gene in the midline in *ptc* mutants rescues the funneling phenotype. (A): The abundance of Robo1 is not affected in *ptc* embryos. Wild-type and *ptc* mutant embryos were stained for the expression of Robo1. The abundance of Robo1 is unaffected in longitudinal tracts in 15 hpf *ptc* mutant embryos. The presence of Robo in commissures in *ptc* is due to the inappropriate midline crossing of Robo-positive longitudinal tracts and not from commissural tracts. Scale bar: 8 μm. (B, C, D): *ptc* mutant embryos transiently expressing *slit* at the midline between 11 and 13 hours of development from a *UAS-slit* transgene driven by the midline driver *sim-*GAL4 were stained for Slit (B) and Fas II (C, D). These panels show that the transient midline expression of *slit* restores Slit in the midline and in tracts, albeit at a lower concentration (B), and rescues the funneling phenotype (B-C). It also improves the fasciculation of longitudinal tracts compared to non-rescued *ptc* embryos (D), although it is possible that this improvement in fasciculation is contributed by the widening of the tracts in rescue embryos. Note that the midline crossing of the medial tract in *ptc* is *slit-*independent and expected to be present in rescued embryos. Scale bar: 8 μm. (E): Developmental stages/time, with key axonal guidance events (upper) and temperature shifts of *ptc/ptc; UAS-slit/sim-GAL4* embryos (lower). Maternal *slit* gets exhausted and zygotic *slit* transcription begins between 4–6 hours of development [7]. The (+), or the (-) signs indicate rescue/no rescue in corresponding upshifts. The thick bar between 11–13 hpf of development corresponds to the period just as endogenous *slit* expression begins to decay in the posterior region. It also indicates the rescue effect by the expression of the *slit* gene between these two time points. (F): Wild-type and *ptc* mutant embryos were stained with an antibody against Sim. Note that even at 15 hpf Sim expression is mostly unaffected with only disorganization of cells and gaps along the midline. The distance between L-L tracts across the midline (in μm with standard deviation) with Fas II staining in rescued embryos are as follows: anterior region: 26+/-0.9; posterior region: 26.8+/-1.0. N = 6 embryos per set. The difference in the distance between tracts in the rescued embryos to wild-type was not statistically significant, but it was in the posterior end between the rescued and *ptc* mutant embryos (P<0.001, student *t-*test). This indicates a rescue of the narrowing of tracts in *ptc* mutant embryos with the transient expression of *slit* at the midline. DAB-histochemistry was used for color development of embryo staining. Scale bar: 8 μm.

The staining of older stage *ptc* mutant embryos for Slit also revealed the A-P funneling defect of axon tracts in *ptc* mutant embryos (Fig 2F and 2G). In these embryos, the gradual disappearance of Slit followed a gradual funneling of tracts (Fig 2F). In those instances where the disappearance of Slit was dispersed or random, the narrowing of tracts followed the loss or reduction of Slit in the midline and in tracts (Fig 2G). These results suggest that Slit is essential for maintaining the position of tracts away from the midline in a parallel trajectory in older stage embryos. Unlike the loss of Slit expression, the abundance of Robo1 in tracts in *ptc* mutant embryos was uniform along the A-P axis of the nerve cord (Fig 3A; n = 30 embryos; N = 3 experiments), indicating that the limiting factor for the tracts positioning in *ptc* was Slit and not Robo. Since the Comm protein down-regulates Robo1 only in commissural tracts at the midline, the three Robo1-positive longitudinal tracts (Medial or M, Intermediate or I, and lateral or L tracts) that crossed the midline at AC and PC in *ptc* mutants had Robo1 in tracts at the midline in these regions. Note that the inappropriate midline crossing defects of longitudinal tracts in *ptc* mutants did not show any specific differences between anterior and posterior regions and were more or less the same in all segments. These results indicate that the funneling defect and the midline-crossing defect are independent of each other. It is also consistent with the fact that the midline-crossing defect is due to the misspecification of NBs and neurons and are *ptc-*dependent but *slit-*independent [20].

Next I sought to determine if the loss of Slit expression in older *ptc* mutant embryos is responsible for the funneling defect and separate it from axon guidance defect that occurs equally in all segments. A UAS-*slit* transgene and the midline driver *single-minded (sim)-GAL4* were introduced to a *ptc* null mutant background and the *slit* transgene was expressed only in the midline at different time points during development. A transient expression of Slit in the midline was induced in embryos raised at 16.5 ^0^C by shifting them to 22 ^0^C (GAL4 does not or very weakly induces UAS-linked transgenes in 16.5 ^0^C but induces at higher levels in 22 ^0^C or above). Induction between 11 and 13 hpf (Fig 3E) resulted in Slit expression at the midline as well as its presence in axon tracts (Fig 3B). This was sufficient to rescue the funneling phenotype of longitudinal tracts in *ptc* mutant embryos (Fig 3B–3E). The rescue was seen in 81%+/-9% of embryos (n = 30 embryos per experiment; N = 3 experiments). A shift between 4–13 hpf also rescued the funneling phenotype (91%+/-12%, n = 20, N = 3), the later shift at 15 hpf, however, did not rescue it (n = 30; N = 3). Measurement of the distance between L-L tracts across the midline in rescued embryos showed that the distance was the same in the anterior and the posterior region. These results indicate that Slit has a direct role in maintaining the position of tracts along the midline, in addition to the initial growth cone guidance [see also ref. 13].

Moreover, the midline expression of the *slit* gene in *ptc* mutant embryos improved the fasciculation defects as well (Fig 3D). While the tracts still crossed the midline, which is Slit-independent, individual tracts could be seen in rescued embryos as opposed to in non-rescued *ptc* mutant embryos where the tracts are mostly de-fasciculated and frayed (Fig 3D). These results suggest that Slit in tracts maintains the fasciculation of axon bundles in longitudinal connectives.

A previous paper had reported that very few midline glial cells exist in older *ptc* mutant embryos [25]. However, we had previously found that *ptc* mutant embryos had midline cells even at 14 hpf [20]. A re-examination of *ptc* null embryos by staining with an antibody against Sim, a midline marker [26], showed that *ptc* mutant embryos had Sim-positive midline cells except for areas with small gaps and clustering of cells (Fig 3F). One explanation for the discrepancy between these results and that of Hummel et al [25] is that Hummel et al examined an unknown and uncharacterized enhancer-trap line that had failed to complement a *ptc* allele, unlike the results of Merianda et al [20] or shown here, where well-known and well-characterized *ptc* alleles were used. Moreover, the midline analysis in Hummel et al [25] used another uncharacterized enhancer-trap line with a midline expression, which itself might be regulated by Ptc. We used Sim, a well-known regulator of fates of all midline cells [26], in these studies.

While the midline expression of the *slit* transgene in *ptc* mutant embryos rescued the funneling phenotype, it did not rescue axons aberrantly crossing the midline (Fig 3C and 3D). This is because the midline-crossing phenotype is mainly due to the earlier events of misspecification of neurons that send out pioneering axons for these tracts [20] and are *slit-*independent. A midline expression of the *slit* transgene will not rescue this or any segmentation defects. These results argue that the gradual anterior-posterior narrowing of tracts in *ptc* is due to the corresponding gradual loss of Slit expression from the midline and in tracts.

**Fig 4:**
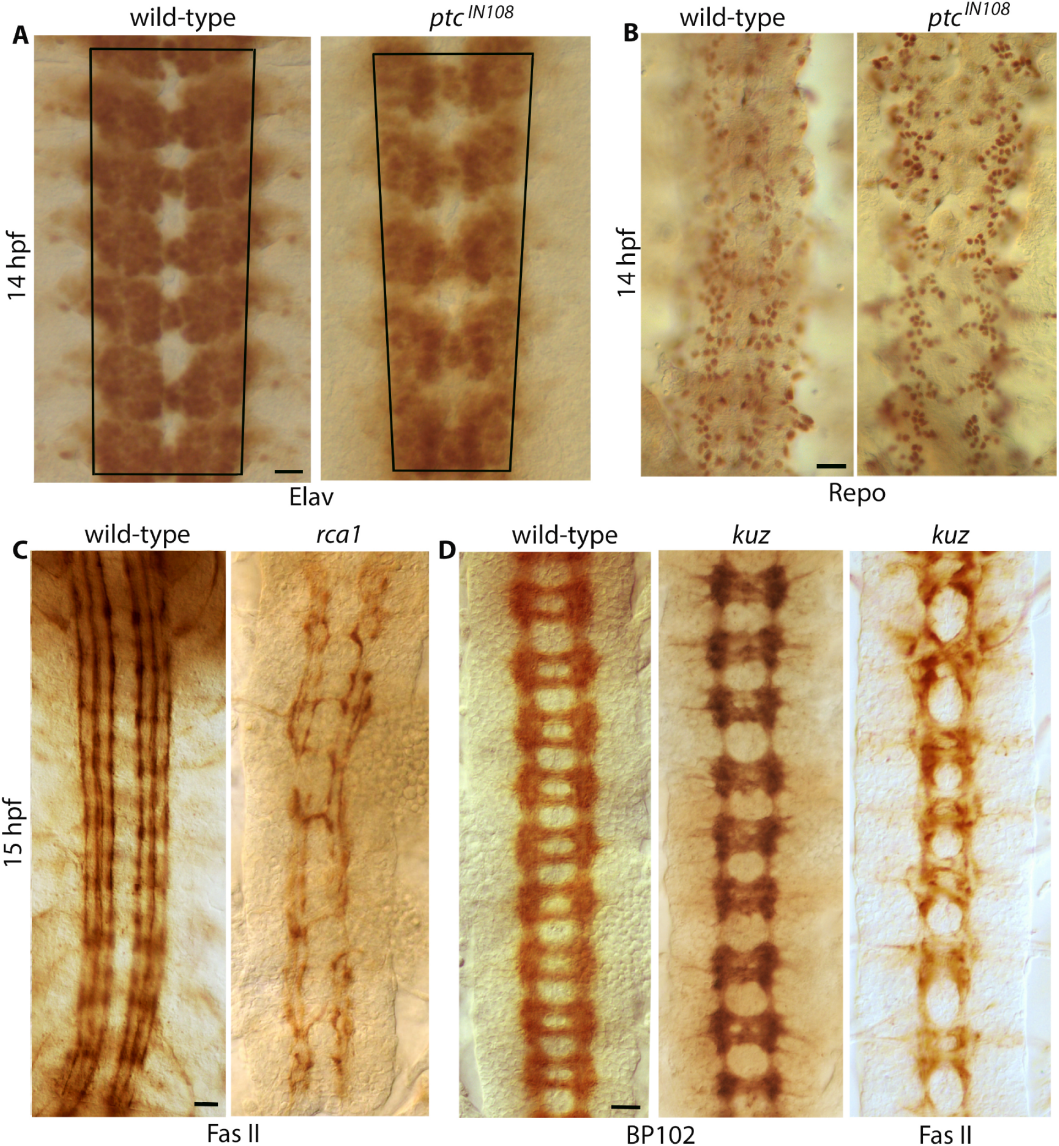
The specificity of the funneling phenotype in *ptc* mutant embryos. (A): Elav-stained wild-type and *ptc* mutant embryos. Note the funneling phenotype in the mutant. The cells in the mutant in the posterior region is much more tightly packed compared to wild-type. A decrease in neuronal number is not evident. Scale bar: 8 μm. (B): Embryos were stained for the expression of Repo, a glial marker. A decrease in glial number is not evident in the mutant. The distribution pattern of glia in the mutant reveals the funneling phenotype. Scale bar: 8 μm. (C): Fas II-stained embryos. In *rca1* mutants, GMCs do not divide but adopt the fate of one of its progeny cells, thus, have a significantly reduced number of neurons. The tracts, however, do not move towards the midline, if anything, they are slightly farther apart. Scale bar: 8 μm. (D): BP102-stained wild-type and *kuzbenian* (*kuz*) mutant embryos (the first two panels) and Fas II-stained *kuz* mutant embryo. Note that *kuz* mutants have significant commissural defects, but they do not show any funneling phenotype or movement of tracts towards the midline. DAB-histochemistry was used for color development of embryo staining. Scale bar: 8 μm.

I also examined if the loss of function for *ptc* progressively reduces the number of neurons in the anterior-posterior direction. Immunostaining of embryos with an antibody against the Elav protein, a pan-neural marker, showed that the number of neurons in *ptc* embryos is not reduced in the anterior-posterior direction (Fig 4A). However, the entire population of neurons in *ptc* embryos appeared to progressively move towards the midline with a tighter packing of neurons per hemisegment in the posterior region. This indicates that neurons, which are closely associated with axon tracts, also move towards the midline in *ptc* embryos. This is likely a secondary effect due to the movement of tracts towards the midline (see Discussion).

Embryos mutant for *ptc* were also examined with Repo, a glial marker. As shown in Fig 4B, there was no significant loss of glial cells, but the location of glial cells had shifted towards the midline in the posterior direction. Since glial cells are attached to the tracts, their movement appears to be the consequence of the movement of tracts. I also examined if a loss of neurons in the nerve cord could cause a shifting of tracts towards the midline. This was done by staining embryos that were mutant for the gene *regulator of cyclin A1* (*rca1*). Rca1 regulates the expression of zygotic *cyclin A* and as in *cyclin A* mutants, in *rca1* mutants also Ganglion Mother Cells (GMCs; these are secondary precursor cells for neurons) do not divide, but adopt the identity of one of their progeny neurons [27, 28]. Thus, their nerve cord possesses significantly fewer neurons and glia than wild-type. As shown in Fig 4C, the longitudinal tracts across the midline in *rca1* were not any closer, instead, they were further away compared to wild-type. This is likely due to the decrease in the number of neurons in *rca1*. An anterior-posterior difference in the position of tracts was also not found in *rca1* mutants. Finally, I examined mutants that appear to have the commissural defects similar to *ptc* to determine if commissural defects could cause the funneling defect. However, the funneling phenotype was not found in such mutants (Fig 4D)[see also ref. 23].

### Loss of function for *comm* shows a Slit-dependent funneling, aberrant positioning and defasciculation of tracts

**Fig 5:**
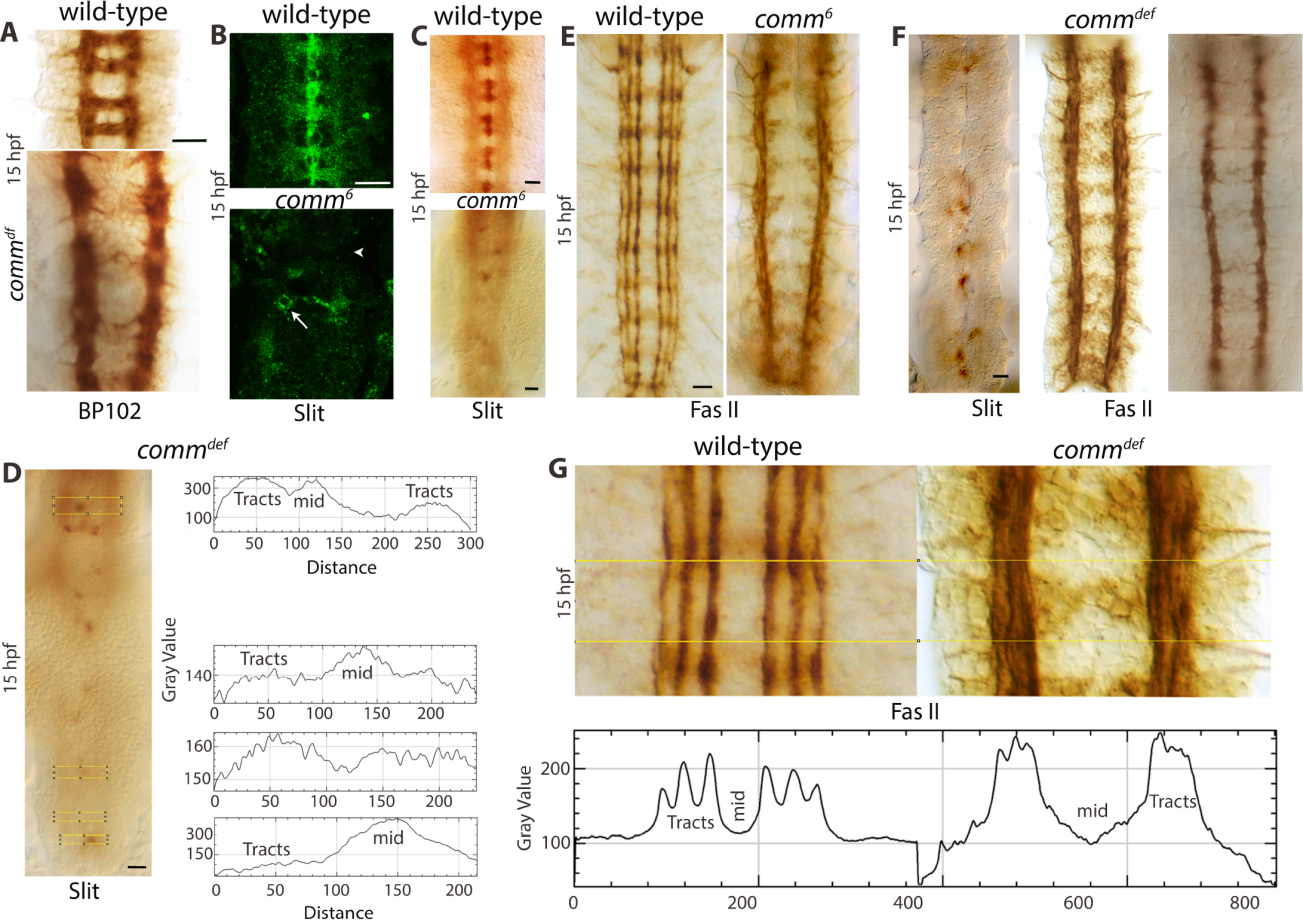
*comm* mutant embryos show a loss of Slit in tracts, the funneling phenotype and defasciculation of tracts. (A): BP102-stained wild-type and *comm*^*deficiency (df)*^ mutant embryos. Only a few commissural axons cross the midline in a random fashion in *comm* mutants. Note the funneling phenotype of longitudinal connectives towards the posterior region in *comm* embryos. Scale bar: 8 μm. (B): Slit-stained wild-type and *comm* mutant embryos, analyzed with confocal microscopy. Note the characteristic midline and commissural staining of Slit in wild-type, and the absence of such a staining in *comm*. In *comm*, Slit-positive midline glial cells are often mislocated (arrow) and tract staining is mostly absent (arrowhead) or seen in intermittent regions. Scale bar: 8 μ. (C): Slit-stained wild-type and *comm* mutant embryos. In *comm*, the abundance of Slit is progressively affected towards the posterior region of the nerve cord. The funneling phenotype of the longitudinal connectives is also seen. About half of the *comm* mutant embryos show this pattern. Scale bar: 8 μm. (D): ImageJ analysis of Slit in a *comm* mutant embryo. The yellow box shows the area of analysis of the Slit profile, presented next to the image. Note the gradual loss of Slit expression from the anterior to the posterior region, as well as the narrowing of the longitudinal connectives. The word mid in ImageJ tracings represents the midline. Scale bar: 8 μm. (E): Fas II-stained wild-type and *comm* mutant embryos. Note the funneling phenotype in *comm* mutant embryos. The tracts are located farther apart and this is primarily due to the midline defect in *comm* embryos (see Fig 6 and Table 1). Scale bar: 8 μm. (F): Slit, Fas II and BP102-stained *comm* mutant embryos, respectively. Note the presence of Slit throughout the longitudinal connectives, although at a lower abundance and the connectives do not show a noticeable funneling phenotype. The Fas II tracts or the BP102 stained connectives also do not show any significant funneling phenotype in such embryos. About 50% of the *comm* mutant embryos show this pattern. Scale bar: 8 μm. (G): Fas II-stained wild-type and *comm* mutant embryos with ImageJ analysis. The yellow box shows the area of ImageJ analysis with profile shown below the image. M, medial tract; I, intermediate tract; L, lateral tract. Note the fraying of longitudinal tracts in *comm*, also illustrated by the ImageJ analysis, which signifies defasciculation of axon tracts. Scale bar: 8 μm.

A loss of function for *comm* severely affects the formation of commissures, although they still have a few axons extending to the midline (see Fig 5A). This loss of most of the commissures in *comm* is due to the upregulation of Robo in commissural growth cones and therefore their inability to overcome the Slit barrier at the midline [24]. These embryos suffer from a significant loss of Slit in tracts (Fig 5B), indicating that proper commissural structures are required for Slit to travel to the longitudinal tracts [see also ref. 7]. Interestingly, we also noticed that in about 40% of *comm* mutant embryos (N = 60 embryos), Slit was present at higher concentrations in tracts in the anterior region compared to the posterior region (Fig 5C and 5D). This corresponded with a higher expression of Slit in the midline glial cells in the anterior region compared to the posterior region, where Slit-expression was lower or the *slit-*expressing cells were absent (Fig 5C and 5D). ImageJ analysis of the anterior versus posterior regions of *comm* embryos for Slit staining quantified and confirmed that there was a significant reduction/loss of Slit in longitudinal connectives from the anterior to the posterior region (Fig 5D; see also S1A Fig). As in *ptc* mutant embryos, in *comm* mutant embryos as well, a narrowing of tracts in the anterior-posterior direction was observed (Fig 5C and 5D). This defect corresponded with the gradual reduction in the levels of Slit in tracts.

Fas II staining of *comm* mutant embryos also revealed that while the longitudinal connectives/tracts were farther apart in *comm* mutant embryos, about twice the distance of wild-type (see Table 1; S1 Data), the tracts were funneling down towards the posterior end (Fig 5E; 52%+/-4%). This funneling defect was seen also with BP102 (Fig 5A) and Slit staining (Fig 5C and 5D). On the other hand, in about 50% of the embryos (N = 60) where the tracts had low levels of Slit all along the tracts, no recognizable funneling phenotype was seen (Fig 5F). These results, therefore, provide a second mutational data showing a similar funneling defect as in *ptc* embryos and its correlation with the decrease in the abundance of Slit in tracts.

Each of the longitudinal tracts occupies a specific position along the nerve cord. Slit in tracts appears to regulate the distance between these tracts. While Comm is present and is required in the midline and in commissural tracts spanning the midline, it is not present in longitudinal connectives [23, 24]. In *comm* mutants, however, the positioning of longitudinal tracts was aberrant with intermingling of tracts with each other. Additionally, the tracts were defasciculated (Fig 5E, 5F and 5G, see also S1B Fig). ImageJ analysis of such Fas II-stained nerve cord from *comm* mutant embryos illustrate the intermingling and defasciculation of tracts (Fig 5G; see also S1B Fig).

**Fig 6:**
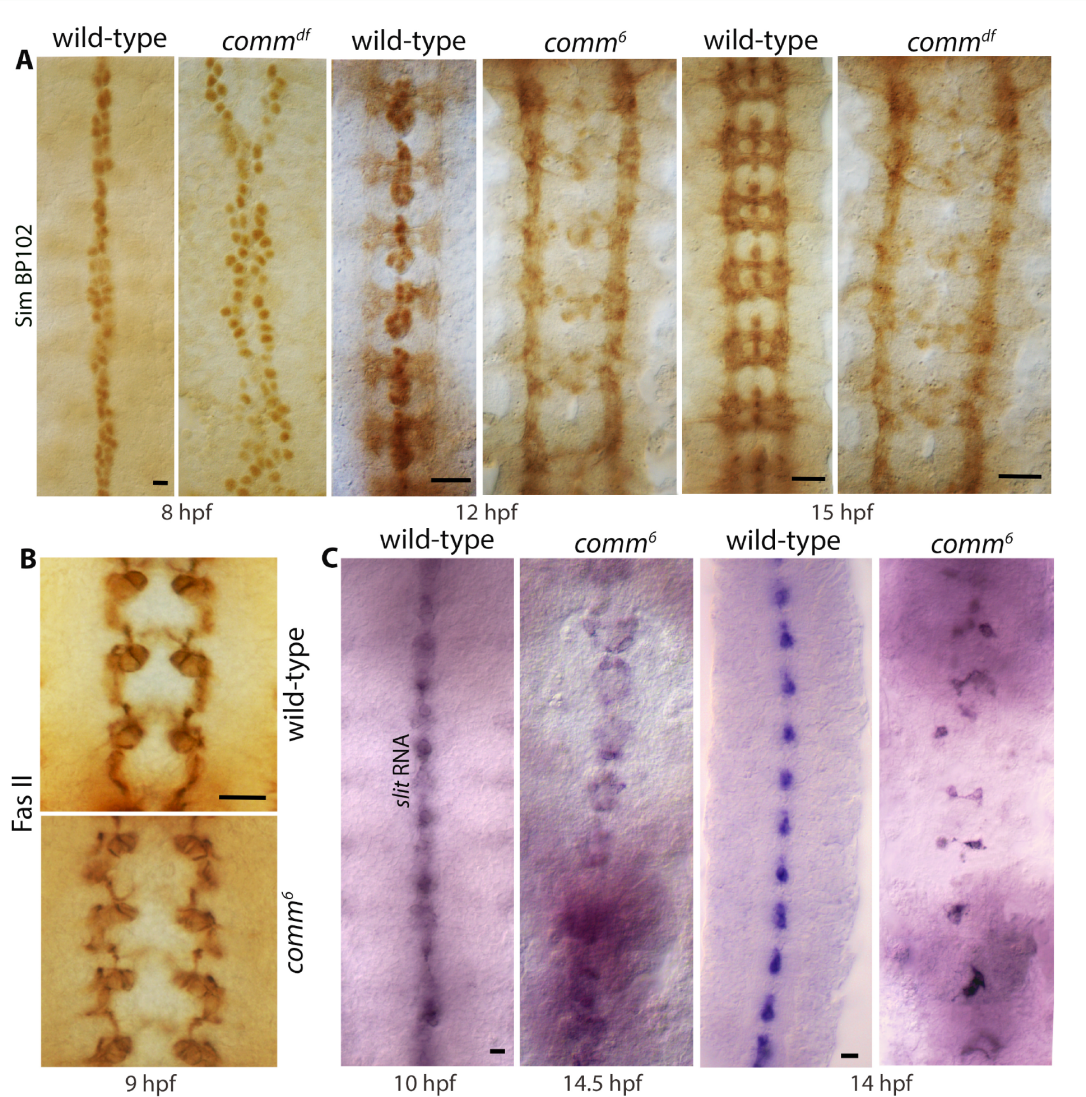
Loss of function for *comm* shows significant midline defects. (A): Sim and BP102-double stained embryos. Note the disorganization of the midline, which begins during early neurogenesis and is discernible by 8 hpf. Sim has a midline expression, BP102 has a ladder-like pattern. Scale bar: 8 μm. (B): Fas II stained wild-type and *comm* mutant embryos. The initial position of the medial tract in *comm* is farther apart than in wild-type even at an early stage. Scale bar: 20 μm. (C): Whole-mount RNA in situ for *slit* in wild-type and *comm* mutant embryos. Note that Slit-positive midline glial cells fail to organize into a single file of cells along the midline in *comm* mutants. Scale bar: 8 μm.

The longitudinal tracts in *comm* mutants are positioned farther away from the midline compared to wild-type. A loss of cohesion between hemineuromeres due to the absence of commissures could be the reason. However, it appears that the midline is severely affected in *comm* mutant embryos [29, 30]. Staining of *comm* mutant embryos with an antibody against Sim (Fig 6A), Fas II (Fig 6B) and *sli* RNA expression analysis (Fig 6C) showed that the midline was severely affected. The midline cells in *comm* failed to form a single line as in wild-type, but dispersed into two disorganized lines even at an early age (Fig 6A). This early origin of the midline defect in *comm* affecting the position of tracts was indicated by the finding that in *comm* the initial position of the medial tract was placed farther away compared to wild-type (Fig 6B, see also S2 Fig). This result argues against the possibility that the commissural positioning defect in *comm* is due to a loss of cohesion from the absence of commissural structures. Despite their aberrant position, tracts in *comm* indeed moved closer towards the midline in the posterior region, which strengthens the argument that Slit has a maintenance function, both the position of tracts as well as their fasciculation.

### Slit in tracts interacts with Robo1 in tracts to maintain the position of longitudinal tracts

**Fig 7:**
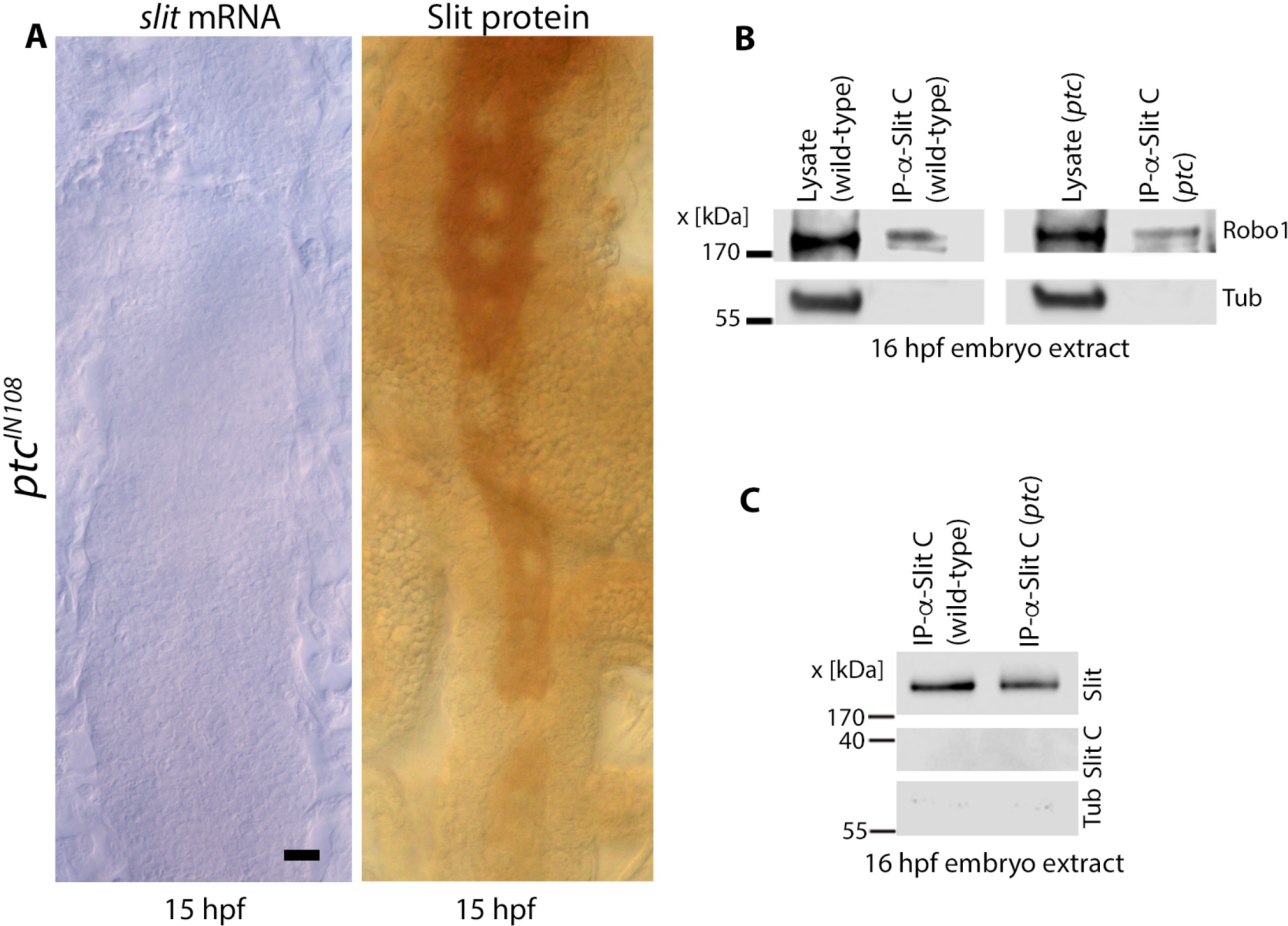
Slit in tracts interacts with Robo in tracts. (A): *slit* mRNA and the Slit protein in 16 hpf *ptc* mutant embryos. There is a complete loss of *slit* transcription and the Slit protein in the midline by 16 hpf but the tracts still have residual Slit in a decreasing gradient from the anterior to the posterior direction. This expression pattern corresponds to the loss of *slit* mRNA from the midline in the anterior-posterior direction (see also Fig 2F and 2G). This anterior to the posterior gradient pattern of Slit in tracts also corresponds to the progressive funneling of tracts. Scale bar: 8 μm. (B): Pull-down assay for Robo1 with anti-Slit-C in extracts from 16 hpf old *ptc* mutant embryos when Slit (and Robo1) is present only in tracts. (C): The IP from the above pull-down assay using anti-Slit-C was examined for the presence of Slit in the immunoprecipitate. Only the full-length Slit could be seen; the Slit-C fragment is not usually detectable in Westerns (see Fig 8). Tubulin was used as a loading control.

In *ptc* mutant embryos that were ~16 hpf or older did not have *slit* transcription or the Slit protein in the midline (Fig 7A). But, these embryos still had the Slit protein in axon tracts, with the highest amount in the anterior region of the nerve cord. The position of tracts in the anterior region was more or less normal (Fig 7A). With the Slit in tracts gradually decreasing towards the posterior region, the tracts were also getting progressively narrower (Fig 7A). By taking advantage of the expression pattern of Slit in these >16 hpf *ptc* mutant embryos, I sought to determine if the Slit in tracts physically interacts with the Robo in tracts, perhaps to regulate inter-tract spacing. If the Slit in tracts physically and locally interacts with the Robo1 in tracts, we should be able to pull down Robo1 with Slit in extracts from older stage *ptc* mutant embryos. Embryo extracts from >16 hpf old wild-type and *ptc* mutants were prepared and subjected to immunoprecipitation using an antibody against Slit-C (which recognizes the full-length Slit as well). The immunoprecipitated proteins were resolved on a polyacrylamide gel and probed for the presence of Robo1. If Robo1 is pulled down by Slit, given that Slit in these older stage *ptc* embryos is present only in tracts but not in the midline, the interaction between Slit and Robo1 must be occurring in tracts. As shown in Fig 7B, Robo1 was indeed pulled down by immunoprecipitation with anti-Slit in the extract from ~16 hpf *ptc* mutant embryos.

**Fig 8:**
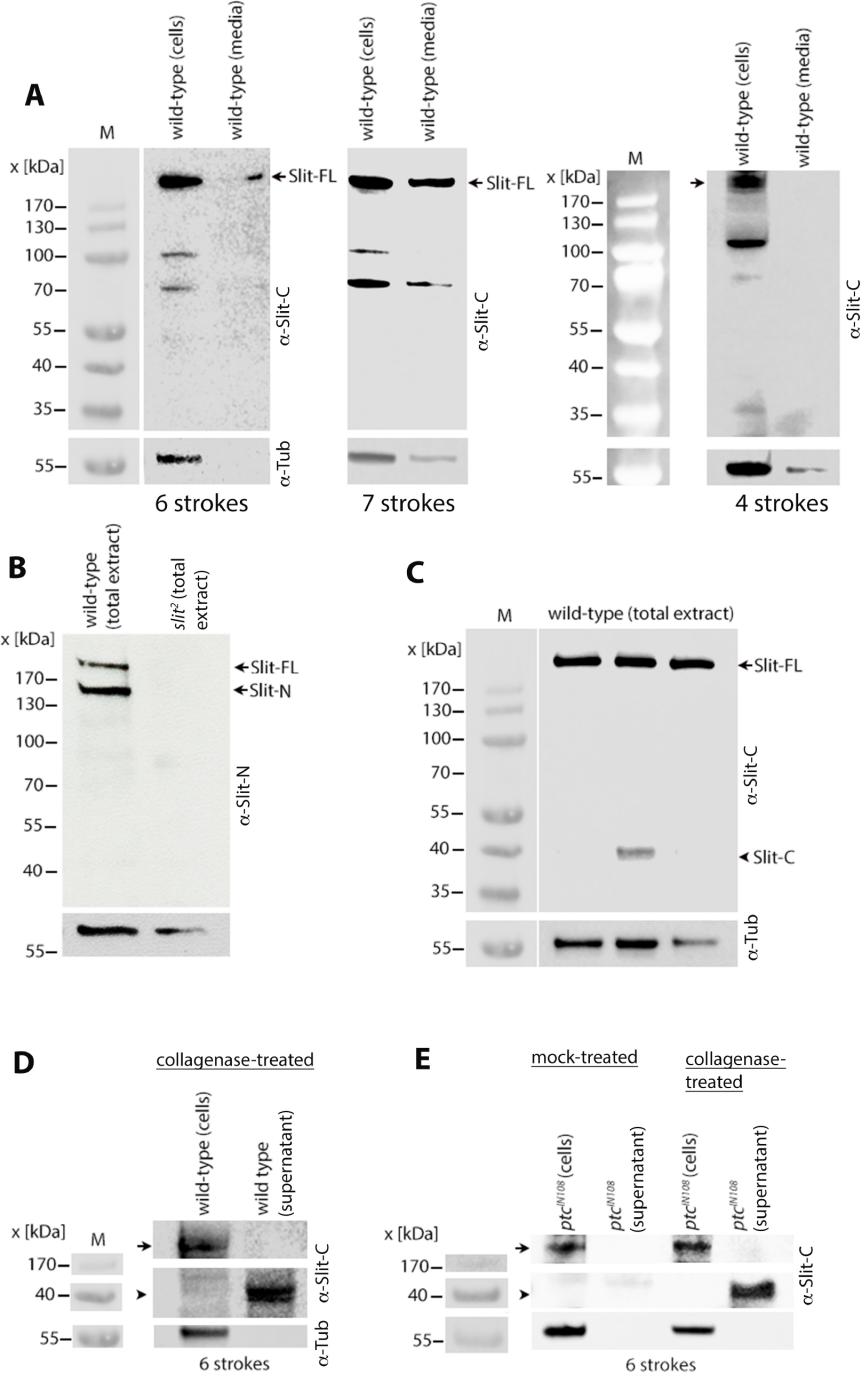
Full-length Slit and Slit-N are loosely associated with the ECM, whereas Slit-C is tightly bound to the ECM. Extracts were derived from ~16 hours old embryos and were prepared under identical conditions unless otherwise noted. Tubulin was used as a loading control. (A): Full-length Slit is detected in the media only with >4 mechanical strokes of embryonic cells in a Dounce homogenizer. Slit-C is not detected in these samples either in the cells-pellet extracts or in the media. (B, C): The Slit-N peptide is readily detected in total embryo extracts with anti-Slit-N (B), whereas the Slit-C fragment is rarely detected in total embryo extracts with anti-Slit-C (C). Both antibodies readily detect full-length Slit. (D): Slit-C is readily detected in the supernatant of embryonic cells treated with collagenase but is not detected without the collagenase treatment. (E): Slit-C is also present in axon tracts as indicated by its presence in the collagenase-treated supernatant in *ptc* mutant embryos.

The Slit protein is processed into an N-terminal fragment of~150 kDa Slit-N and a C-terminal fragment of ~40 kDa Slit-C. It has been proposed that Slit-N interacts with Robo to guide longitudinal tracts, whereas Slit-C interacts with PlexinA1 to mediate commissural tracts [15, 16]. The results presented in Fig 7B show that an antibody raised against Slit-C pulls down Robo1. This would suggest that Slit-C interacts with Robo1. However, since this antibody against Slit-C also recognizes full-length Slit, it may be that the antibody pulls down Robo1 through full-length Slit. Therefore, I examined if the same immunoprecipitate complex also has the full-length Slit. As shown in Fig 7C, the immunoprecipitated complex contained full-length Slit, indicating that the full-length Slit interacts with Robo1 (see also Fig 8). However, no Slit-C was detected in the immunoprecipitate (Fig 7C; Slit-C migrates around 40 kDa, see also Fig 8).

### Slit, Slit-N and Slit-C are not present in the extracellular milieu but associated with the ECM

I have recently developed a procedure to detect proteins that are secreted in vivo in embryos (see Materials and Methods). This method involves dissociating cells from embryos in M3 cell culture media with a Dounce homogenizer and analyzing the media and the cellular pellet for the protein in question using Western analysis. When the dissociation of cells was done using 6 strokes with the loose-fitting pestle in the Dounce homogenizer, the full-length Slit was readily recovered in the media (Fig 8A). With 7 strokes, there appeared to be cell-lysis, as indicated by the presence of Tubulin in the media (Fig 8A), although this Tubulin could also be from contaminating or broken axons. Dissociating cells with 4 strokes yielded no Slit in the media but only in the pellet (Fig 8A), indicating that Slit is not freely present in the milieu. The extra mechanical dissociation force caused by 6 strokes appears to release the externalized Slit protein from the ECM into the media.

The Slit-N peptide could also be readily detected in embryo extracts with an antibody raised against Slit-N (Fig 8B), indicating that Slit-N is also loosely associated with the ECM. However, Slit-C was only rarely detected in embryo extracts (Fig 8C) or in the media with 4–7 strokes (Fig 8A). Thus, Slit-C appears to be more tightly bound to ECM with its epitope perhaps buried within the ECM and not easily accessible/detectable. Only when the ECM somehow gets disrupted Slit-C could be detected in total extracts (Fig 8C).

To determine if Slit-C is tightly bound to ECM, I subjected the mechanically dissociated embryonic cells (6 strokes) to collagenase treatment (see Materials and Methods). Collagenase treatment is expected to disrupt the ECM. The supernatant was then subjected to Western analysis. I found that the treatment with collagenase consistently released Slit-C, and was readily detected in Western blots (Fig 8D). This result argues that disruption of the ECM with collagenase releases Slit-C. Since in older stage *ptc* mutant embryos (>15 hours old), the Slit protein is absent from the midline, but still present in tracts, I sought to determine if the tracts also contain Slit-C. Indeed, cells derived from >15 hours old *ptc* mutant embryos generated Slit-C but only when treated with collagenase (Fig 8E).

### Robo1 interacts physically with full-length Slit and Slit-N but not with Slit-C

**Fig 9:**
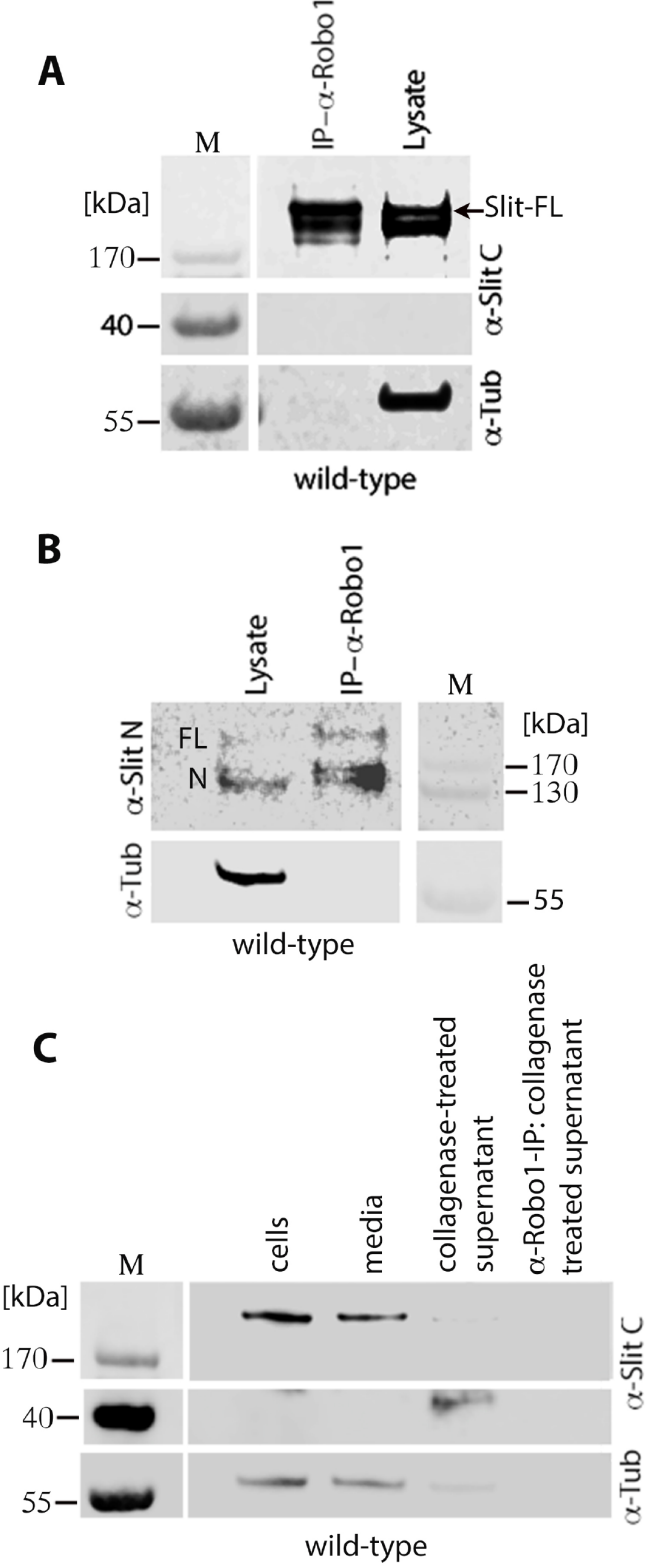
Slit and Slit-N but not Slit-C binds to Robo1. Extracts were derived from ~16 hpf wild-type embryos and were prepared using identical conditions unless otherwise noted. Tubulin was used as a loading control. (A): Immunoprecipitation of the embryonic extract with anti-Robo1 antibody pulls down Slit but not Slit-C. (B): Immunoprecipitation of the embryonic extract with anti-Robo1 antibody pulls down both Slit and Slit-N. (C): Immunoprecipitation of the collagenase-treated embryonic extract with anti-Robo1 does not pull-down Slit-C.

Anti-Slit-C pulls down Robo1 as well as Slit (Fig 7B and 7C) indicating that Slit-C pulls down the Slit-Robo1 complex. To further confirm this, I immunoprecipitated total cell extract from ~15 hours old wild-type embryos with anti-Robo1 antibody and determined if it pulls down full-length Slit. As shown in Fig 9A, anti-Robo1 pulled down full-length Slit, indicating that Robo1 physically interacts with full-length Slit. However, Slit-C was not detected in the immunoprecipitate (Fig 9A). That Robo1 also complexes with Slit-N is indicated by the result that anti-Robo1 pulls down Slit-N (Fig 9B). Since the absence of Slit-C in the immunoprecipitate could be due to the buried nature of Slit-C in the ECM, I treated total cell extracts with collagenase and then performed IP of this collagenase-treated extract with anti-Robo1. The IP was then analyzed by Western analysis with the anti-Slit-C antibody. As shown in Fig 9C, analysis of the IP with anti-Slit-C showed that Robo1 does not pull down Slit-C. Thus, Slit-C is unlikely to interact with Robo1, although it is possible that collagenase treatment somehow altered Slit-C such that it could not interact with Robo1.

## Discussion

**Fig 10:**
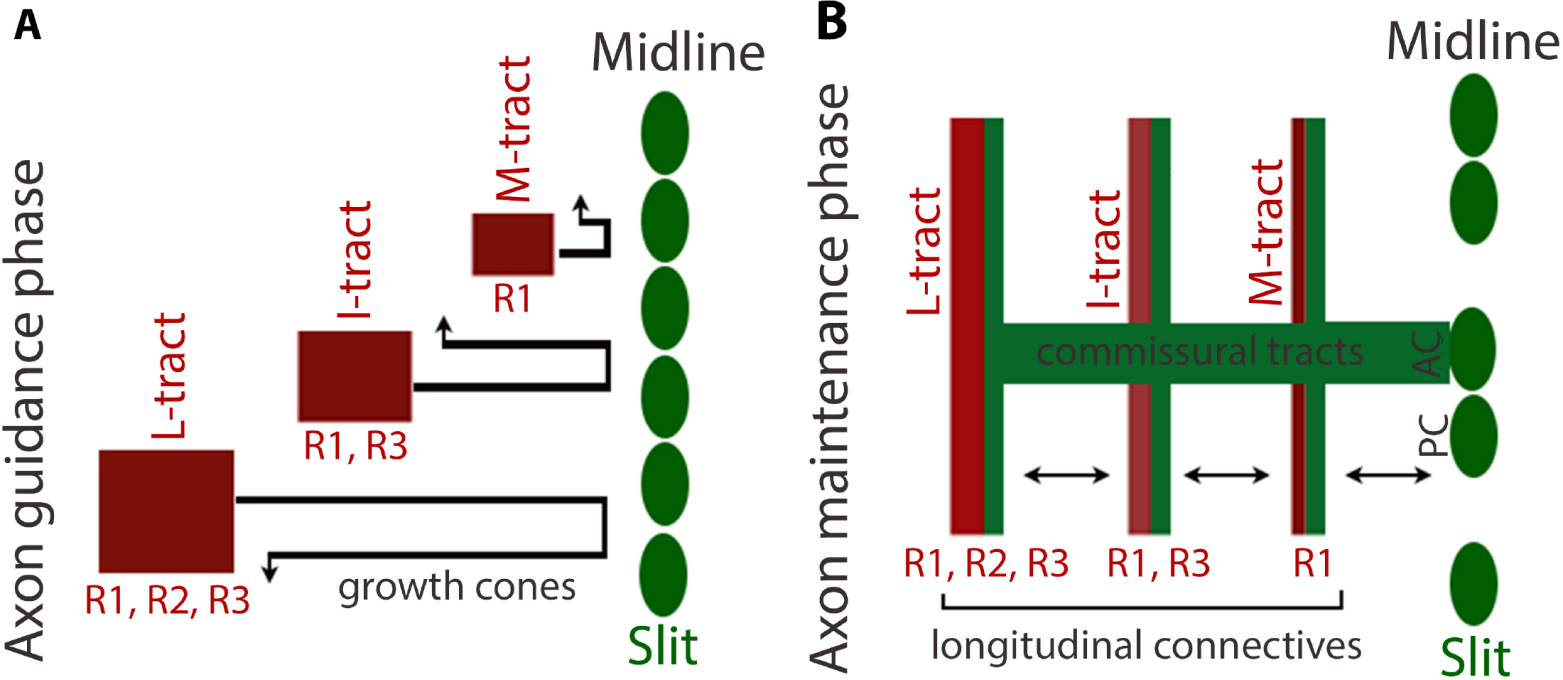
Slit-Robo signaling during early and late embryogenesis and within axon tracts. (A): The Slit-Robo signaling during early neurogenesis specifies the initial position of longitudinal tracts. Robo1 is present in growth cones that give rise to the M-tract, Robo1, and Robo3 in growth cones of the I-tract, and Robo1, Robo2, and Robo3 in growth cones of the L-tract. These growth cones project towards the midline where they encounter Slit. The combined strength of Robo-Slit interaction specifies the lateral position of each tract: the L-tract growth cones, because of the highest amounts of Robo proteins and, the strongest Robo-Slit effect, occupy the lateral-most position. The M-tract growth cones, because of the lowest levels of Robo (only Robo1), occupy a position closest to the midline. The I-tract growth cones, with Robo greater than the M but less than the L, occupy a middle position. (B): The Slit-Robo signaling maintains axon tracts in their position from the midline and between tracts after their guidance. The secreted Slit from the midline is transported along the commissural tracts to the longitudinal connectives (which are composed of both the longitudinal tracts and commissural tracts). Slit then gets distributed along or adjacent to the M, I, and L-tracts. The Slit and Slit-N are bound loosely to the ECM of commissural tracts and interacts with Robo in the adjacent longitudinal tracts. This Robo-Slit interaction in tracts maintains the fasciculation of individual axons within tracts and the position of tracts between each other and from the midline. By maintaining inter-tract position within the connectives, Slit-Robo also maintains their position away from the midline as tracts are unable to move from their position. These results suggest that the tracts have a default position and that default position is at the midline.

The hardwiring of the nervous system, in general, constitutes axon pathfinding, axon fasciculation, dendritic arborization, and synaptic connectivity/plasticity. But, do organisms need to actively maintain hardwiring in the CNS, and if so, how do they accomplish this? Would it involve axon guidance molecules? The results described in this paper show that the position, as well as the fasciculation of tracts, are actively maintained within the CNS. Signaling by Slit-Robo appears to have a significant role in these events since loss of Slit later during neurogenesis causes loss of positioning and fasciculation of tracts [see also ref. 13]. These results also illuminate how Slit signaling could mediate these functions, which also sheds light on the significance of the presence of Slit in axon tracts. A combinatorial amount of Robo proteins interacting with Slit at the midline and then at the tracts appears to mediate the position and maintenance of tracts and their fasciculation (Fig 10).

### Slit-Robo signaling within axon tracts in the maintenance of tracts

The results shown here argue that Slit in connectives maintains the position of tracts in reference to the midline and axonal fasciculation within each tract-bundle. This appears to occur via Slit interacting with Robo proteins locally in the tracts (Fig 7B). These conclusions are reached based on the following findings. First, the pattern of decay of Slit expression and the funneling phenotype in *ptc* or *comm* embryos. A progressive loss of Slit in tracts in an anterior-posterior direction was accompanied by a progressive narrowing of axon tracts (Figs 1B, 1D, 2F, 5A, 5C, 5D, 5E and 7A). In mutant embryos where the loss of Slit was interspersed, the narrowing of tracts corresponded to those regions with loss or reduction of Slit in tracts (Figs 2G and 7A). Second, in *ptc* mutant embryos of ~16 hours of age or older, the midline expression of Slit was completely lost, but the anterior region of the nerve cord still had a high enough amounts of Slit in tracts (Fig 7A). In such embryos, the tracts were maintained at their more or less correct position in the anterior region, but not in the posterior region where the midline Slit was long gone and the abundance of Slit in tracts was much reduced. The position of tracts was also shifted progressively like a funnel in these embryos (Fig 7A). Third, while the tracts in *comm* mutant embryos were placed farther away from the midline compared to wild-type or *ptc*, which appears to be due to midline defects (Fig 6), whenever there was a loss of Slit in tracts, such embryos showed a narrowing of tracts (Fig 5). Thus, a continuous presence of Slit at the midline serves as a source for Slit in connectives (see Fig 10), and the Slit in connectives appears to be essential for maintaining the position of tracts. Since immunoprecipitation of extracts with anti-Slit-C from these older stage embryos pulled down Robo1, Slit and Robo1 must physically interact with one another in tracts of the connectives.

The funneling phenotype in *ptc* is unlikely due to a secondary, patterning defect or a general impact of defective commissures. Because, a loss of function for *ptc* affects patterning and other CNS defects (segmentation and NB specification defects) equally in all hemisegments, these defects are unlikely to cause the highly specific funneling phenotype. Would the aberrant commissures in *ptc* pull the longitudinal tracts mechanically closer to the midline? Again, all of the commissures are equally affected in *ptc* embryos and this occurs well before any defect in Slit expression occurs. The funneling phenotype is seen only in regions where Slit expression is lost (mostly towards the caudal end). Furthermore, mutations that affect commissures similar to the defects in *ptc*, do not cause the funneling phenotype seen in *ptc* (Fig 4D). There does not appear to be any loss of neurons in *ptc* embryos specific to the posterior region either, only that cells are more tightly packed towards the posterior end of the nerve cord (Fig 4A and 4B). Also, even a significant reduction in the number of neurons in the nerve cord does not cause longitudinal tracts to move towards the midline (Fig 4C). Finally, the rescue experiment shows that the funneling phenotype in *ptc* could be rescued without rescuing the guidance or commissural defects (Fig 3C, 3D and 3E), effectively separating the two and showing that axon guidance or commissural defects would not necessarily cause the funneling phenotype. This is consistent with our previous finding that axon guidance defects in *ptc* (midline-crossing of longitudinal tracts) are *slit-*independent but *ptc-*dependent, and mediated by the misspecification of neurons that pioneer these tracts [20]. Finally, a loss of Slit in tracts, but not in the midline in *comm* mutants causes longitudinal tracts to move towards the midline, towards each other and also become de-fasciculated (Fig 5), supporting the possibility that tracts Slit prevents both de-fasciculation and inappropriate movement of tracts towards the midline (see also S1 Fig).

In older *ptc* mutant embryos, both neurons and glia exhibited the funneling phenotype. Neurons and glia are an integral part of axon bundles, like grapes on a wine or beads on a rope, and keeping the tracts/bundles in a specific location within the nerve cord means keeping those neurons and the structure itself in place. The entire nerve cord in that sense is one continuous unit. Whatever that acts on tracts possibly affects the entire structure. Thus, the entire structure in *ptc* in the caudal region seems to move towards the midline, causing cells in the posterior region to pack more tightly. This phenomenon in *ptc* or *comm* may be analogous to the phenomenon of nerve cord retraction—the tracts and the entire nerve cord retracts and progressively gets shorter and shorter beginning with ~13 hours of development. This nerve cord retraction in all likelihood is mediated by shortening of tracts, pulling the entire structure towards the anterior end, thus leading to the condensation of the entire nerve cord.

Since Robo1 co-immunoprecipitates only Slit and Slit-N, but not Slit-C (B, C, Fig 9A and 9B), Robo1 must interact with Slit and Slit-N, but not with Slit-C. Also, Slit and Slit-N appear to be loosely associated with the ECM (Fig 8A and 8B), whereas Slit-C is likely buried or tightly bound to ECM, perhaps inaccessible to Robo. These results are consistent with the finding that while Slit-N interacts with Robo to guide longitudinal tracts Slit-C interacts with PlexinA1 to regulate commissural guidance [15, 16]. The results also show that the full-length Slit also interacts with Robo1, not just Slit-N, thus potentially mediate axon guidance. Whether Slit versus Slit-N further refines Slit-Robo signaling is not known. The presence of Slit-C in tracts in *ptc* mutant embryos likely reflects perhaps its role in regulating guidance and maintenance of commissural axons.

### Slit-Robo signaling in axon guidance versus axon maintenance

A following scenario emerges on how Slit-Robo combination could mediate axon guidance to define lateral positioning of longitudinal tracts, and later on regulate tracts position and fasciculation. Soon after its formation, a neuron generates neurites, which repeatedly extend and retract from the cell body. Out of several of these neurites, only one becomes an axon. The axonal growth cone starts to sample its environment while extending from the cell body towards the midline. In the fly embryonic nerve cord, the growth cones of longitudinal axons have Robo proteins, but the three Robo proteins are differentially expressed in growth cones of different tracts. Thus, growth cones of the medial tract have only Robo1, the intermediate tract have Robo1 and Robo3, and the lateral tract have Robo1, Robo2, and Robo3 (Fig 10A). Thus, the lateral tract growth cones have the highest amounts of Robo proteins, the intermediate tract has the next highest and the medial tract has the lowest Robo. During early neurogenesis, these growth cones will explore their environment and when they reach the midline, the interaction between Slit at the midline and the combined Robo in growth cones specifies the initial position of axons in a Robo-concentration dependent manner. Because the highest combined amounts of Robo is in lateral growth cones, they occupy the lateral-most position. The lowest amounts of Robo in the medial growth cones will specify their position closest to the midline. In this scenario, increasing the levels of Slit at the midline will not alter the eventual positioning of tracts, but the availability of Robo proteins/their saturation would. This was indicated by the previous findings that gain of function for Robo2 and Robo3 proteins in tracts shifted them away from the midline [4–6] but the over-expression of Slit in the midline had no effect [3].

During the maintenance phase (Fig 10B) when the growth cones of these tracts have successfully fasciculated with each other, a Slit-Robo interaction can occur only in tracts since growth cones are no longer exploring their environment but are stably fasciculated. But a Slit-Robo1 interaction indeed occurs in tracts (Fig 7B and 7C) where both are present. As shown previously [7, 13], and in this report, Slit from the midline gets transported to the longitudinal connectives along the commissural tracts (Fig 10B). The longitudinal connectives have both the longitudinal axon tracts as well as the commissural tracts that extend along the longitudinal tracts after crossing the midline (Fig 10B). The Slit in commissural tracts, possibly embedded in the ECM, must then interact with Robo in longitudinal tracts to maintain the fasciculation of individual axons within each tract, as well as their position between and from the midline (Fig 10B). The exact mechanism of Slit transport from the midline along the commissures to the tracts is not known. But, secretion of Slit is essential since loss of function for *mummy*, a gene that encodes an enzyme in the protein glycosylation pathway, and glycosylates Slit, prevents Slit secretion and causes an absence of Slit in tracts [13]. Consistent with a role for Slit in tracts, mutants in *mummy* show defasciculation and loss of distance between tracts and from the midline. Does Slit form a morphogen-gradient? Given our results, it appears unlikely, but perhaps a gradient of a modified Slit (and thus, unrecognized by the antibody) or some type of a functional gradient is still a possibility. Our result that Slit-Robo signaling contributes to the maintenance of hardwiring of the CNS has far-reaching implications in deciphering the hardwired neural circuitry networks and their functional aspects in normal and abnormal conditions across organisms.

## Materials and methods

### Fly stocks and genetics

Standard genetics were used [31]. The following fly stocks were used: *ptc*^*IN108*^, and *ptc*^*H84*^ which are phenotypically null alleles, *ptc*^*deficiency*^ [Df (2R) ED1742], *slit*^*2*^, *robo1*^*4*^, *robo1 deficiency* [Df (2R) BSC786], a *comm* deficiency [Df(3L)BSC845], a deficiency for *kuz* [Df(2R)IR52a-d] and a deficiency for *Sdc* [Df(2R) 48] were from the Bloomington Stock center, *comm*^*5*^, *comm*^*6*^ were from Mark Seeger and Guy Tear, *UAS-slit* was from the Goodman lab, *rca1* from Nipam Patel, and *sim-GAL4 (*on the 3^rd^ chromosome) was from Steve Crews. For wild-type, Canton-S and Oregon-R flies were used. Mutant chromosomes were balanced using *green fluorescent protein* (*GFP*)-marked chromosomes (twi-GFP, CyO, Kr-GFP, CyO) to enable the selection of homozygous mutant embryos. Mutant embryos were further identified using their mutant phenotypes in other tissues and lineages.

### Rescue experiment

To determine if the expression of the *slit* gene in the midline in *ptc* mutants restores the axon tract position defects in *ptc* mutants, *UAS-slit* was introduced to *ptc* mutant background and induced in the midline using *sim-GAL4*. The UASxGAL4 induction system is sensitive to temperature since the GAL4 protein is not active at lower temperatures, especially at 16.5 ^0^C. Therefore, embryos from the above “rescue” cross were allowed to develop at 16.5 ^0^C for various developmental time points (see Fig 3E) before shifting to 22 ^0^C, where the embryos were allowed to develop until they were fixed for staining or down-shifted to 16.5 ^0^C and kept until they were fixed. The embryos were then examined for the distribution of Slit and axon guidance defects. Embryos kept at 16.5 ^0^C grow about 40% slower compared to embryos at 22 ^0^C. The timings and stages were then compensated by taking this difference into account while representing the results in Fig 3E. Moreover, when the embryos were shifted to 22 ^0^C, they were ascertained for their correct stages of development by visualizing a subset of them under the microscope after permeabilizing with Halocarbon oil [see ref. 31]. Thus, for example, an 11 hpf embryo shift would mean that embryos were collected for 15 min at 16.5 ^0^C, they were then allowed to develop at 16.5 ^0^C for an equivalent of 11 hours at 22 ^0^C, which is about 15.5 hours of time at 16.5 ^0^C. To positively ascertain the developmental stages of embryos before shifting, about 50 embryos were removed from the collection at 15.5 hpf, immersed in Halocarbon oil, which allows to monitor the stages of development clearly, and the stages were confirmed. To be certain, only those batches of embryos that showed the correct developmental stages corresponding to development at 22 ^0^C were shifted to 22 ^0^C.

### Immunohistochemistry and RNA whole mount in situ

Immunochemistry was performed as previously described [7, 13, 32]. Monoclonal and or polyclonal antibodies against the following proteins were used at the indicated concentrations: Fas II (1:20, mouse monoclonal 1D4, Developmental Studies Hybridoma Bank (DSHB), Slit-C (1:20, mouse, C555.6D, DHSB), Slit-N (1:4000), Robo1 (1:3, mouse, 13C9, DHSB), Elav (1:4, mouse, 9F8A9, DHSB), Repo (1:4, 8D12, DHSB). The monoclonal antibody BP102 recognizes an uncharacterized epitope on axons was used at 1:4 (AB_528099, DHSB). For confocal microscopy, secondary antibodies conjugated to Cy5 (rabbit, 1:400, Invitrogen, A10523), fluorescein isothiocyanate (mouse, 1:50, Invitrogen, 62–6511), Alexa Fluor 488 (rabbit or mouse, 1:300, Invitrogen, A-11008 or A-11001), or Alexa Fluor 647 (rabbit or mouse, 1:300, Invitrogen, A-21245 or A-21236) were used. For light microscopy, secondary antibodies conjugated to alkaline phosphatase (AP; rabbit, 1:200, Pierce, 31341) or horseradish peroxidase (HRP; rabbit, 1:200, Pierce, 31460) were used. Alkaline phosphatase was detected using 5-Bromo-4-chloro-3-indolyl-phosphate and nitro blue tetrazolium (Promega, S3771). HRP was detected with diamino-benzidine (Sigma, D4418). Whole-mount RNA in situ hybridization for *slit* was done as described previously using a Digoxigenin-labeled *slit* probe synthesized by PCR [20, 32] and the color reaction was developed by AP reaction. Different genotypes were identified using appropriate markers and phenotypes.

### Western analysis

Western blot analysis was done as previously described [7, 13, 32]. Briefly, about 30 embryos of the specified age were collected and used for protein extraction. Embryos were collected on apple-juice agar plates, transferred to a mesh-wire basket, washed with running water, and the mutant embryos were selected (absence of green excitation for GFP) using a Zeiss microscope equipped with an Ultra-Violet (UV) light. The embryos were lysed in 40 μL extraction buffer (0.15 M NaCl, 0.02 M Tris pH = 7.5, 0.001M EDTA, 0.001 M MgCl2, 1% Triton-X-100 and 1X Protease Inhibitor Cocktail) using a sonicator for one minute on ice in a 1.5 mL Eppendorf vial. A hand-held, sonicator (Fisher Scientific) equipped with a disposable pestle (Fisher Scientific) was used for sonication. The lysates were centrifuged for 5 minutes at 13,000 rpm in a microfuge (Beckman). The supernatant was collected and 10 μL of 4X Laemelli buffer and 1.5 μL of the reducing agent (Invitrogen) were added, boiled in water for 10 minutes and cooled on ice. About 15 embryos-equivalent amounts were loaded per lane on a 4–12% pre-made SDS-PAGE gel (Invitrogen). Two different primary antibodies recognizing Slit were used: Slit-N, which recognizes the N-terminal portion (1: 50000) [7]; Slit-C, which recognizes the C-terminal portion (mouse, C555.6D, DHSB, 1:100) [3]. For Robo1 Westerns, mouse monoclonal 13C9 (DHSB) was used at 1:40 [32]. The chemiluminescent reaction kit (Millipore) was used to detect signals. The blots were re-probed with an antibody against Tubulin (1:4000, Abcam) to determine the loading control.

### Co-immunoprecipitation experiment

About 200 wild-type and *ptc* mutant embryos, aged 16 hours of development, were homogenized in 37.5 μL of ice-cold lysis buffer [50mM HEPES (pH 7.2), 100mM NaCl, 1mM MgCl_2_, 1mM CaCl_2_, and 1% NP-40]. The lysates were incubated on ice for 30 minutes, centrifuged at 15,000X g for 30 minutes at 4°C. 30 μL of the supernatant was used as starting material for each IP reaction using the Catch and Release v2.0 Reverse Immunoprecipitation System (Millipore #17500). The columns were washed with 1X Wash buffer (Millipore) thrice (2000X g, 20 seconds) and the IP reaction was set up by directly adding these ingredients to the column in the following order: 1X 435 μL of the wash buffer, 30 μL of the cell lysate, 25 μL of the antibody against Slit-C and 10 μL of the antibody capture affinity ligand. For immunoprecipitating with anti-Robo1, the IP reaction was set up as follows: 405 μL of the wash buffer, 30 μL of the cell lysate, 50 μL of the monoclonal antibody against Robo1 and 10 μL of the antibody capture affinity ligand. The columns were then incubated overnight at 4 ^0^C. The flow through was collected and the columns were washed three times with 1X Wash buffer (2000Xg, 20 seconds), and eluted with 60 μL of PBS-based denaturing elution buffer (2000Xg, 20 seconds). For equalizing salt concentration between the lysate and the IP samples, 8 μL of the lysate was added to 8 μL of the denaturing elution buffer (Millipore), and 8 μL of the lysis buffer was added to 8 μL of the IP (which is in the denaturing elution buffer). The proteins were then separated on a 4–12% SDS-PAGE and immunoblotted with a monoclonal against Robo1 (1:40, DHSB), against Slit-C (1:100) or against Slit-N (1:40000). Signal detection was by a chemiluminescent ECL reaction kit. The blots were re-probed with an antibody against Tubulin (1:4000, Abcam) to determine the loading control.

### Analysis of Slit in tracts, outside of the midline

I have recently developed a method by which one could detect secreted proteins within and outside of cells without having to culture cells. Briefly, about 75 embryos were collected in 500 μL of M3 insect cell culture medium. They were transferred to a 1.5 mL Dounce homogenizer. The cells from these embryos were dissociated using the looser fitting pestle and different number of strokes (without twisting the pestle) during homogenization. Cells quickly dissociate in M3 media, which is confirmed by visualizing an aliquot of the homogenate under a microscope. The homogenates were transferred to 1.5 mL Eppendorf tube and centrifuged at 4000x g for 5 min. The supernatant was collected into a Vivaspin 500 (Sartorius)(molecular weight cutoff 100 kDa) concentrator and microfuged at 15000x g for 17 min. The resulting ~30 μL of the media were then subjected to Western analysis for Slit. The pellet in the Eppendorf tube was washed once by gently re-suspending the cells in 500 μL of M3 media and microfuging at 4000x g for 5 min. The supernatant was discarded. The pellets (containing the cells) were then lysed using the Lysis buffer, and subjected to Western analysis for Slit.

### Collagenase treatment to release Slit-C from the ECM

About 75 wild-type or *ptc* mutant embryos were collected directly in 500 μL of M3 insect cell culture medium. The cells were dissociated in a Dounce homogenizer (using the looser fitting pestle) by using 6 strokes (without twisting the pestle) during homogenization (see Fig 8). The homogenates were transferred to 1.5 mL Eppendorf tube and centrifuged at 4000x g for 5 min. The supernatant was removed and the cell-pellet was re-suspended in 100 μL of PBS, centrifuged at 4000x g for 5 min. The supernatant was discarded, and the pellet was re-suspended in 20 μL of PBS. Collagenase type VII (2 μL of 10 mg/mL solution) was added to these cells and incubated at room temperature for 20 min. The cells were then centrifuged at 4000x g for 7 min, and the supernatant was collected for Western analysis. The cells-pellet was then re-suspended in lysis buffer, homogenized and then subjected to Western analysis for Slit. As a control, another batch of embryos was processed similarly but without adding collagenase. The pellets were then lysed using the Lysis buffer, and subjected to Western analysis for Slit.

### Immunoprecipitation of collagenase treated-samples

About 75 wild-type embryos were collected directly in 500 μL of M3 insect cell culture medium. The cells were dissociated in a Dounce homogenizer by using 6 strokes. The homogenates were transferred to 1.5 mL Eppendorf tube and centrifuged at 4000x g for 5 min. The media was removed and concentrated using the Vivaspin concentrator (see above). The cells-pellet was re-suspended in 40 μL of the Lysis buffer (see above under Co-Immunoprecipitation experiment). Collagenase (4 μL of 10 mg/mL solution) was added to these cells and incubated at room temperature for 20 min. The cells were then centrifuged at 4000x g for 7 min, and the supernatant was collected and 30 μL of this supernatant was subjected to immunoprecipitation using anti-Robo1 antibody and the IP was processed as described above (under co-immunoprecipitation experiment). Western analysis was done using anti-Slit-C antibody.

## Supporting information

S1 Fig Absence/reduction of Slit in tracts and its effect on the inter-tract spacing of longitudinal tracts in *comm* mutant embryos. (A, B): Wild-type and *comm* mutant embryos stained with an antibody raised against Slit-C. Note the high levels of Slit in the midline and in tracts in wild-type but only in the midline in *comm*. The Slit in the midline and in tracts was quantified using ImageJ analysis, which shows that in *comm*, unlike in wild-type, there is little or no Slit in tracts. (B) Fas II-stained wild-type and *comm* mutant embryos with ImageJ analysis. Note that longitudinal tracts in *comm* are not organized into discreet bundles, an indication of axon defasciculation. M, medial tract; I, intermediate tract; L, lateral tract. Scale bar: 8 μm. (C): Fas II-stained wild-type and *Sdc* embryos. Note that the medial tract crosses the midline (arrow) in *Sdc*, but the remaining tracts are minimally affected and the medial tract midline crossing is seen only in a few segments. ImageJ analysis indicates that the longitudinal tracts in *Sdc* are organized into discreet structures unlike in *comm* or *ptc* mutant embryos. Scale bar: 8 μm.
(PDF)

S2 Fig The position of tracts is farther apart in *comm* mutant embryos. Wild-type and *comm* embryos were stainend for BP102.
(PDF)

S1 Data Raw data for Table 1 and other measurements in the manuscript. (XLSX)

S2 Data (DOCX)
