# Supplementary figures and images for: Post-guidance signaling by extracellular matrix-associated Slit/Slit-N maintains fasciculation and position of axon tracts in the nerve cord
Source: PLoS Genet. 2017 Nov 20;13(11):e1007094. doi: 10.1371/journal.pgen.1007094 (PMC5714384; doi:10.1371/journal.pgen.1007094)

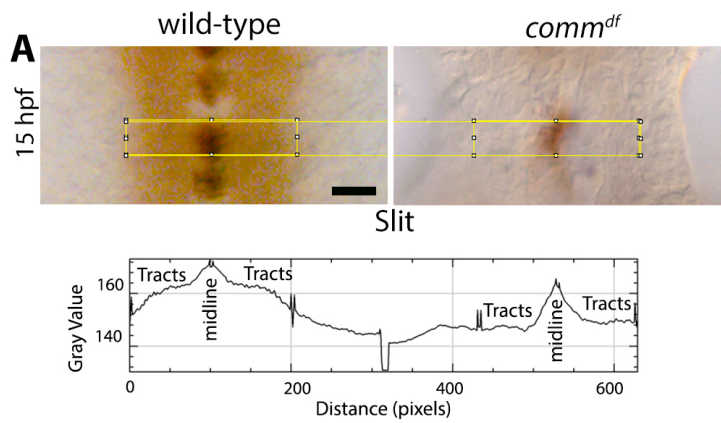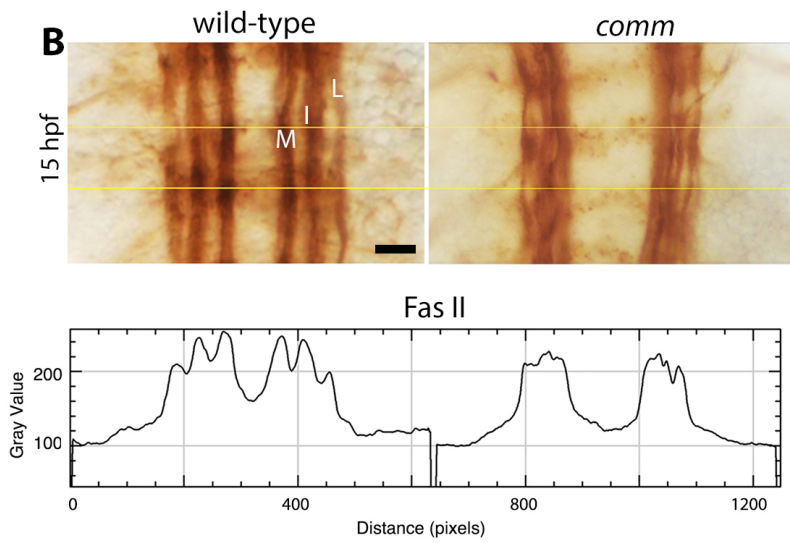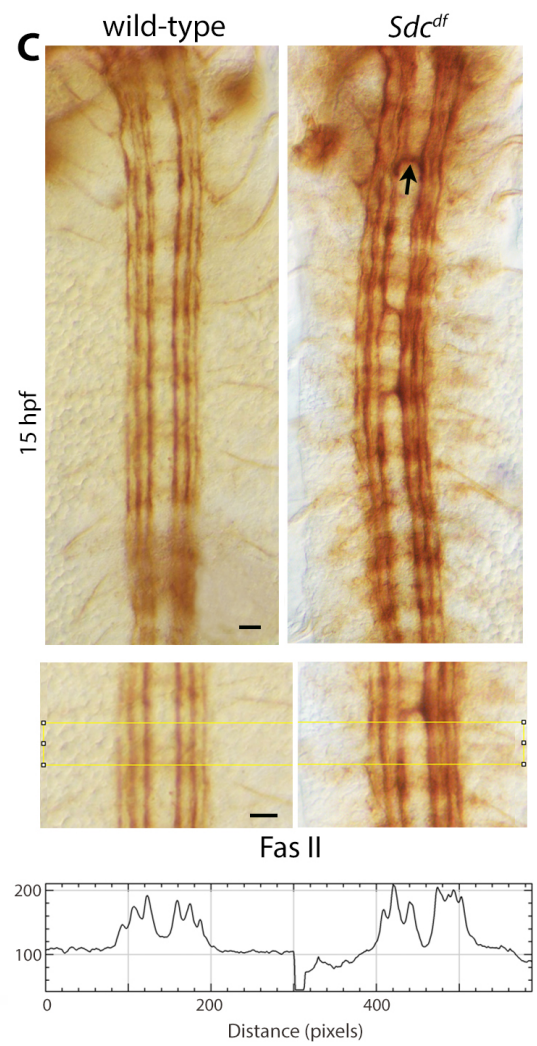

Supplement: S1 Fig — (A, B): Wild-type and comm mutant embryos stained with an antibody raised against Slit-C. Note the high levels of Slit in the midline and in tracts in wild-type but only in the midline in comm. The Slit in the midline and in tracts was quantified using ImageJ analysis, which shows that in comm, unlike in wild-type, there is little or no Slit in tracts. (B) Fas II-stained wild-type and comm mutant embryos with ImageJ analysis. Note that longitudinal tracts in comm are not organized into discreet bundles, an indication of axon defasciculation. M, medial tract; I, intermediate tract; L, lateral tract. Scale bar: 8 μm. (C): Fas II-stained wild-type and Sdc embryos. Note that the medial tract crosses the midline (arrow) in Sdc, but the remaining tracts are minimally affected and the medial tract midline crossing is seen only in a few segments. ImageJ analysis indicates that the longitudinal tracts in Sdc are organized into discreet structures unlike in comm or ptc mutant embryos. Scale bar: 8 μm. (PDF) [file pgen.1007094.s001.pdf]

9.5 hpf

wild-type

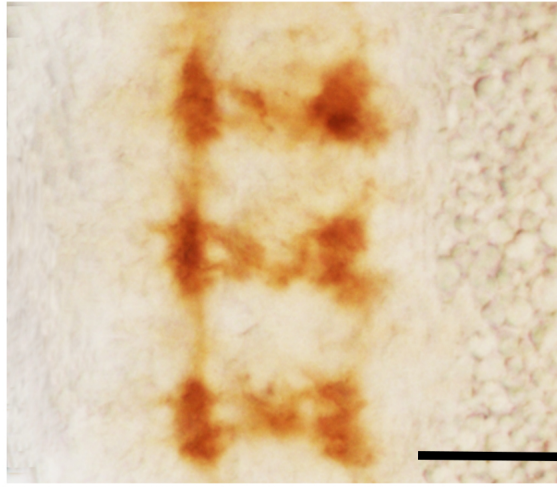

comm

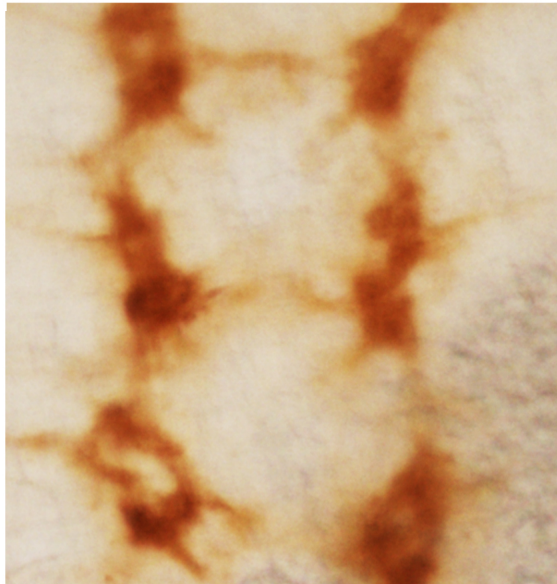

BP102

Supplement: S2 Fig — Wild-type and comm embryos were stainend for BP102. (PDF) [file pgen.1007094.s002.pdf]
