# Supplementary material for: Post-guidance signaling by extracellular matrix-associated Slit/Slit-N maintains fasciculation and position of axon tracts in the nerve cord
Source: PLoS Genet. 2017 Nov 20;13(11):e1007094. doi: 10.1371/journal.pgen.1007094 (PMC5714384; doi:10.1371/journal.pgen.1007094)
Supplement: S2 Data — (DOCX) [file pgen.1007094.s004.docx]

**Supplementary Information**

**Post-guidance signaling by extracellular matrix-associated Slit/Slit-N maintains fasciculation and position of axon tracts in the nerve cord**

**Axon maintenance by Slit and Slit-N**

Krishna Moorthi Bhat

Department of Neuroscience and Cell Biology, University of Texas Medical Branch School of Medicine, Galveston, Texas, United States of America

Email: [kmbhat@utmb.edu](mailto:kmbhat@utmb.edu)


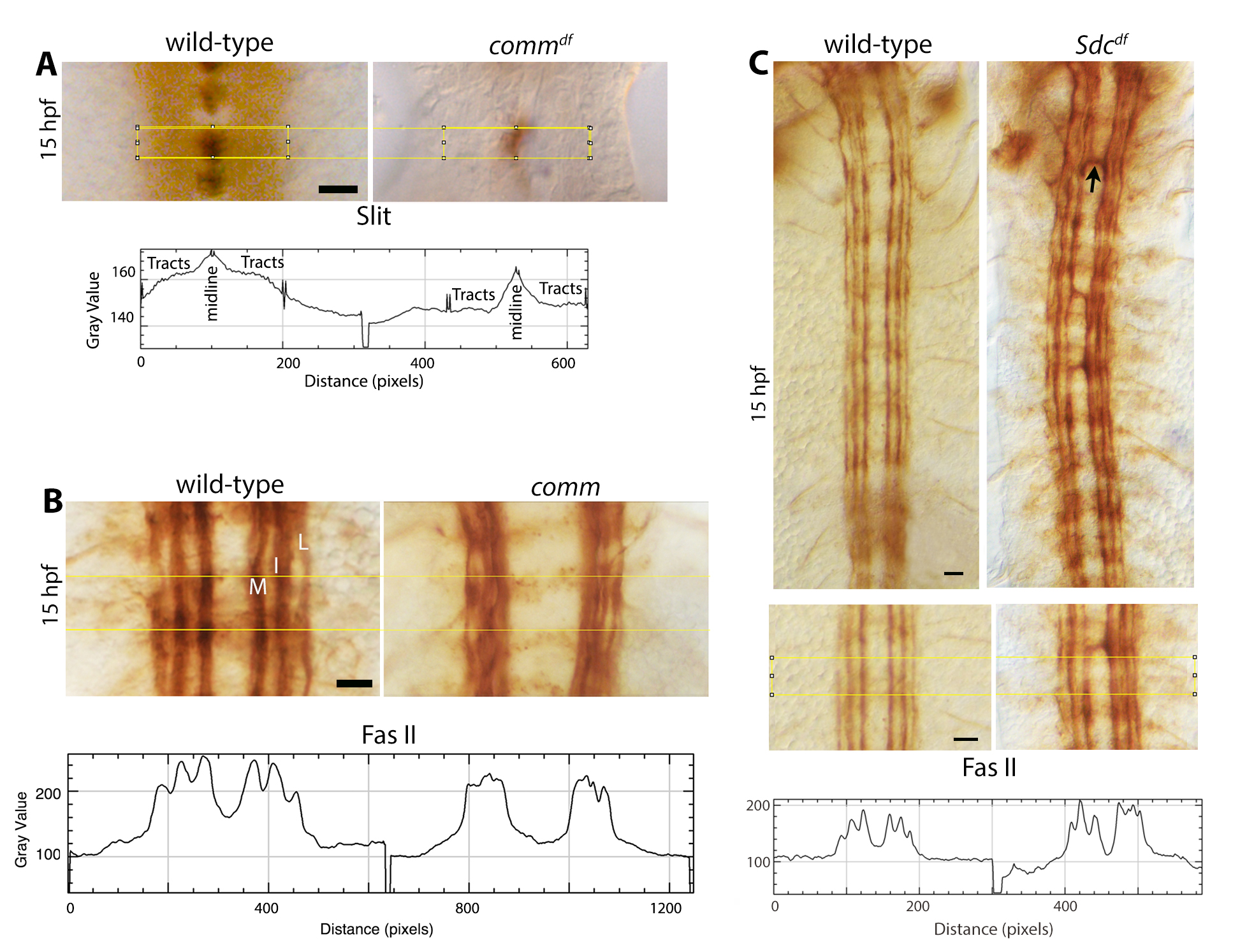


**Figure S1: Absence/reduction of Slit in tracts and its effect on the inter-tract spacing of longitudinal tracts in *comm* mutant embryos.**

(A, B): Wild-type and *comm* mutant embryos stained with an antibody raised against Slit-C. Note the high levels of Slit in the midline and in tracts in wild-type but only in the midline in *comm*. The Slit in the midline and in tracts was quantified using ImageJ analysis, which shows that in *comm,* unlike in wild-type, there is no Slit in tracts. (B) Fas II-stained wild-type and *comm* mutant embryos with ImageJ analysis. Note that longitudinal tracts in *comm* are not organized into discreet bundles, an indication of axon defasciculation. M, medial tract; I, intermediate tract; L, lateral tract. Scale bar: 8 μm.

(C): Fas II-stained wild-type and *Sdc* embryos. Note that the medial tract crosses the midline (arrow) in *Sdc*, but the remaining tracts are minimally affected and the medial tract midline crossing is seen only in a few segments. ImageJ analysis indicates that the longitudinal tracts in *Sdc* are organized into discreet structures unlike in *comm* or *ptc* mutants. Scale bar: 8 μm.


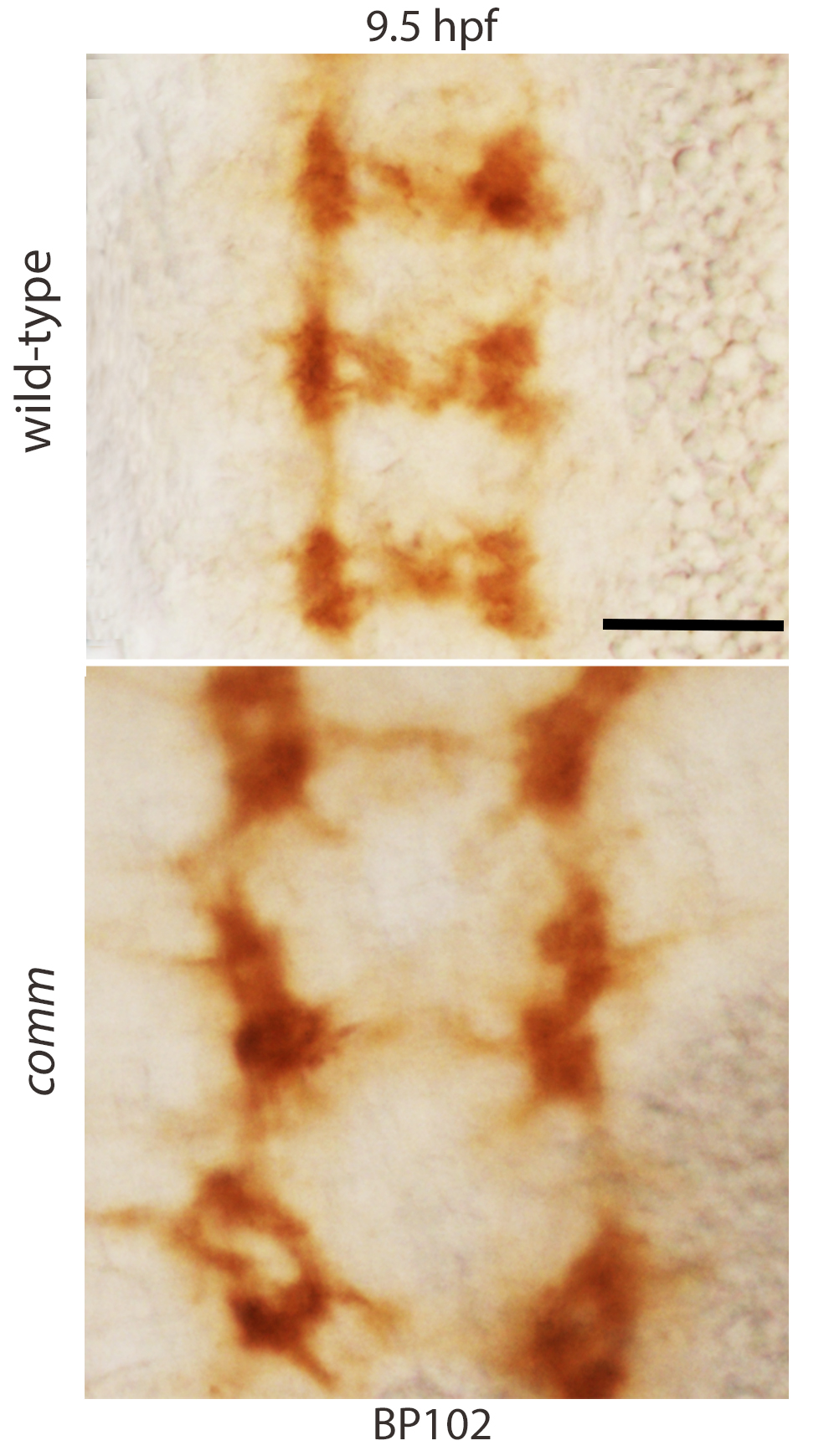


**Figure S2: Position of tracts are positioned farther apart in *comm* mutant embryos.**

Wild-type and *comm* embryoswere staiend for BP102.
